# Modifications of FLC Physical Properties through Doping with Fe_2_O_3_ Nanoparticles (Part I)

**DOI:** 10.3390/ma14164722

**Published:** 2021-08-21

**Authors:** Sebastian Lalik, Olaf Stefańczyk, Dorota Dardas, Natalia Górska, Shin-ichi Ohkoshi, Monika Marzec

**Affiliations:** 1 Institute of Physics, Jagiellonian University, 30-348 Kraków, Poland; sebastian.lalik@doctoral.uj.edu.pl; 2 Department of Chemistry, School of Science, The University of Tokyo, Tokyo 113-0033, Japan; olaf@chem.s.u-tokyo.ac.jp (O.S.); ohkoshi@chem.s.u-tokyo.ac.jp (S.-i.O.); 3 Institute of Molecular Physics, Polish Academy of Sciences, 60-179 Poznań, Poland; dardas@ifmpan.poznan.pl; 4 Faculty of Chemistry, Jagiellonian University, 30-387 Kraków, Poland; natalia.gorska@uj.edu.pl

**Keywords:** ferroelectric liquid crystal, γ-Fe_2_O_3_ nanoparticles, oleic acid, aggregation, metal-organic nanocomposite

## Abstract

The aim of this paper is to show, by systematic studies, the influence of γ-Fe_2_O_3_ nanoparticles on the physical parameters of the liquid crystalline matrix, exhibiting a ferroelectric phase in a wide temperature range. The detailed research was carried out by using diffraction (PXRD), microscopic (OM, SEM, FCPM, POM), thermal (DSC), optical (TLI), electric and spectroscopic (FTIR) methods. We show that even the smallest concentration of γ-Fe_2_O_3_ nanoparticles largely modifies the parameters of the ferroelectric SmC* phase, such as spontaneous polarization, switching time, tilt angle, rotational viscosity, dispersion anchoring energy coefficient and helix pitch. The admixture also causes a significant reduction in the temperature of phase transitions, broadening the SmA* phase at the expense of the SmC* phase and strong streaking of the texture. We present and explain the non-monotonic modification of these parameters with an increase in the nanoparticle concentration. The influence of oleic acid admixture on these parameters is also widely discussed. We have shown that certain parameters of organic-metal nanocomposites can be controlled by the appropriate amount of metal admixture.

## 1. Introduction

The continuous industry development and various areas of social life cause an increase in the demand for materials with precisely defined physical and chemical properties. Nowadays, the soft matter discipline is not limited to widely understood science of organic materials. In recent years, new branches have emerged, e.g., involving organometallic materials. This group of materials is divided into two classes: (1) with and (2) without the presence of a chemical bond between a metal atom and an organic ligand. The above-mentioned classes of hybrid materials are one of the ways to meet the growing demand for specialized materials. Another way is the chemical synthesis of extremely complex organic materials, including the use of longer alkyl substituents, increasing the number of benzene rings in a rigid core and the presence of heteroatoms (such as S, N) [1,2,3,4]. Another possibility is, for example, to introduce lactate groups into the structure of organic compounds or fabrication of a polymer-dispersed liquid crystal [5,6]. In many cases, the latter approach is very costly, time-consuming and requires multiple intermediates and catalysts. In addition, the yields of the end products may be relatively low and/or the material has to be further purified. Therefore, it seems only natural that creating hybrid materials based on existing ones is a good alternative. However, it should be noted that the chemical synthesis of complex compounds makes it possible to predict some of their properties, for example the modification of phase transition temperatures, while the properties of the created composites’ organic material-metal component are practically unpredictable. One of the reasons is the very poor database of these materials. On the other hand, the possibilities of creating hybrid materials are practically unlimited, starting with the choice of matrix (liquid crystal, polymer, gel, sol, resin, silicone, etc.) and ending with the choice of dispersed material (metal, non-metal, isolator, element, chemical compound). Additionally, the dispersed material can be in various forms: crystal, amorphous material, glass, solution, powder, granules, etc. Thanks to the nanotechnology development, it has become possible to synthesize materials with sizes in the order of several nanometers. This is very important because the size of such objects is usually only a few times larger than the single organic molecule that forms the matrix of such a nanocomposite. The commercial cost of the nanoparticles is usually negligible compared to the organic matrix; however, both the cost and the nanoparticles’ diameter depends on the synthesis method [7].

Soft materials, such as liquid crystals (LC), can only exhibit a ferroelectric phase if they are composed of molecules having a non-zero dipole moment and chiral center. Typically, for a new liquid crystalline material, a chiral center is created during its synthesis. However, the easiest way to obtain the chiral liquid crystal material is to introduce a chiral dopant (even a small amount) into the non-chiral matrix [8]. In the case of doping of liquid crystals, the most important issue is the concentration and type of nanoparticles used. Interestingly, different liquid crystalline phases (nematic, smectics, cholesteric) react in a different way to the same admixture.

Until now, many scientists have been involved in the creation and research of new composite materials based on a liquid crystal matrix doped with various types of nanoparticles. Shiraishi et al. doped two nematic liquid crystals NLC-1 and NLC-2 (differing in dielectric anisotropy) with Pd nanoparticles (ca. 2.5 nm with 1, 5, 10 wt.%), and showed that the highest transmission in the on state is for the smallest impurity concentration, and as the frequency increases, the threshold voltage drops drastically for switching to the off state [9]. Joshi et al. doped ferroelectric liquid crystal KCFLC by ZnO nanoparticles (ca. 7 nm) and showed that the admixture improves the alignment of organic molecules in low concentrations (1 wt.%) and the spontaneous polarization and viscosity increase significantly after doping [10]. In turn, Kumar et al. observed nine-fold photoluminescence intensity improvement after doping the ferroelectric liquid crystal LAHS19 with Au nanoparticles (ca. 15–20 nm) [11]. Prakash et al. observed a memory effect due to charge transfer from LC molecules of FLC 6304 to Au nanoparticles (ca. 15–20 nm) [12]. Li et al. also used the non-synthetic method to modify liquid crystal properties and showed that for the MLC-6609 nematic mixture, after doping with BaTiO_3_ nanoparticles (ca. 30–50 nm), the nematic–isotropic liquid phase transition temperature increases by almost 40 °C, as well as an increase in dielectric and optical anisotropy is observed [13]. By doping antiferroelectric liquid crystal mixture with BaTiO_3_ nanoparticles, we obtained faster molecular switching, a drop in threshold voltage and spontaneous polarization, as a result of modification of the local electric field [14]. In turn, Manepalli et al. showed that the self-organization of ZnO nanoparticles (ca. 61 nm, 1 wt.%) added to 9OBA and 11OBA liquid crystals causes changes in their textures, and transmittance for each functional group increases after 9OBA is doped [15]. Kopčanský et al. doped nematic 6CHBT with Fe_3_O_4_ (0.0001 vol.%), and as a result, a decrease in the specific resistance and an increase in the relaxation time were observed [16]. Manna et al., by doping MHPOBC with BaTiO_3_ nanoparticles (ca. 25 nm, 5 wt.%), significantly decreased the SmA*–SmC* transition temperature and spontaneous polarization, which in turn increases the entropy of the nanocomposite system [17]. Neeraj et al. showed that by doping ferroelectric liquid crystal with Ni nanoparticles (ca. 2–8 nm, 0.5 wt.%), the switching time is shortened, the absorbance in the UV region is improved, the spontaneous polarization and tilt angle (to a small extent) are increased while the conductivity is not significantly changed [18]. The increase in spontaneous polarization, tilt angle and transmittance is explained by the strong interactions between the dipole moments of Ni nanoparticles and surrounding organic molecules in an external electric field. Interestingly, Ni nanoparticles reduced zig-zag structure defects and improved contrast [18]. Roy et al. showed that after doping an antiferroelectric liquid crystal with Na_2_TiO_3_ nanoparticles (ca. 70–80 nm, up to 13 wt.%), the photoluminescence of nanocomposites increased (depending on their concentration) [19]. Additionally, they observed a red shift for the emission bands [19]. Rudzki et al. doped ferroelectric liquid crystal MHOBD4 with ZnO (ca. 50 nm, 0.2 wt.%) and Ag (ca. 100 nm, 0.2 wt.%) nanoparticles [20]. Both admixtures do not affect the molecular tilt angle (the smectic layer’s thickness is constant), but the spontaneous polarization is decreased in both cases while the switching time increased, even by five times [20]. Rožić et al. doped ferroelectric liquid crystal Felix 015/100 with ferromagnetic nanoparticles γ-Fe_2_O_3_ (ca. 18 nm, T_B_ > 380 K), and suggested that the orientation of magnetic nanoparticles is directly coupled to the liquid crystal director field [21]. This coupling concerns the magnetization of nanoparticles and the liquid crystal polarization. These hybrid materials (named soft magnetoelectrics) exhibit both ferromagnetism and ferroelectricity. An additional advantage of these magnetoelectrics is that the external fields well-align nanoparticles by ordering the organic molecules. Moreover, the low dopant concentration (0.14 wt.%) decreased the SmA*–SmC* phase transition temperature. Rožić’s group expressed an important conclusion that the magnetic susceptibility anisotropy follows the temperature evolution of the tilt angle in such hybrid systems. This is direct proof of a certain coupling between the liquid crystal director and the magnetization of nanoparticles. In another paper, Rožić et al. presented that the admixture of Fe_2_O_3_ nanoparticles (ca. 20 nm in toluene; 10, 14 wt.%) to ferroelectric liquid crystal SCE9 suppressed both the Goldstone and soft modes in the SmC* phase [22]. Both modes are suppressed after adding nanoparticles. Likely, a high dopant concentration results in a loss of liquid crystal properties. Due to a very low volume concentration of dopant, changes in material parameters may result from the formation of an electric double layer on the electrode–liquid crystal interface [16]. It should be noted that Fe_2_O_3_ nanoparticles have different magnetic parameters depending on size [23]: very small Fe_2_O_3_ nanoparticles are a superparamagnetic material, while larger-diameter nanoparticles are a ferromagnetic material. In addition, the large diameter distribution of nanoparticles causes the blocking temperatures for each diameter to overlap, and the final result may be impossible to determine.

The influence of the Fe_2_O_3_ nanoparticles on the nematic liquid crystals has been widely studied. Thus, Yadav et al. and Gao et al. obtained a decrease for rise and fall time by doping nematic liquid crystal with Fe_2_O_3_ nanoparticles [24,25], just like Koo et al., who additionally obtained a decrease of threshold voltage and the enhanced dielectric constant, which were explained by the charging of Fe_2_O_3_ nanoparticles in the external field and trapping impurity ions [26]. In turn, Katiyar et al. obtained shorter response times in nanocomposites, which was explained by the trapping ions phenomenon on the surface of TiO_2_ nanoparticles [27]. In turn, by doping to 10OBA and 11OBA with Fe_3_O_4_ nanoparticles (ca. 70–80 nm, 0.5 and 1.0 wt.%), Sivasri et al. obtained a decrease in conductivity, phase transition temperatures, enthalpy changes and transmittance, as well as broadening of the nematic phase temperature range [28].

On the other hand, only a few scientists have reported on the influence of Fe_2_O_3_ nanoparticles on the ferro- or anti-ferro-electric liquid crystals. For example, after doping the ZLI-3654 liquid crystalline mixture with Fe_2_O_3_ nanoparticles (ca. 24 nm, 0.25 and 1.25 × 10^−5^ wt.%), stripe-like domains were observed under crossed polarizers, which were explained by the chain-like alignment of nanoparticles in the LC matrix [29]. In turn, the increase in spontaneous polarization versus applied voltage after doping was explained based on the magnetoelectric coupling resulting from the strong interaction between the nanoparticles’ exchange field (unpaired electron) and the molecular director field [29]. Meanwhile, the rotational viscosity and response time decrease with increasing the voltage in relation to pure ZLI-3654 (modification of the electric field of electric dipoles by a nanoparticle magnetic field). The memory effect was well-observed, especially in the dielectric spectra [29]. Novotná et al. doped the ferroelectric liquid crystal 9HL with γ-Fe_2_O_3_ nanoparticles (ca. 4 nm), which were superparamagnetic at high temperatures, obtaining a super-paramagneto-ferroelectric in the SmC* phase [30]. In turn, Romero-Hasler et al., thanks to the doping of a ferroelectric liquid crystal based on pyrimidine with FeCo nanoparticles (ca. 2–3 nm, 0.53–10.80 wt.%), obtained a multiferroic system in which the magnetic field can be generated by an electric field and vice versa, and the phase transition temperature as well as spontaneous polarization (to a slight extent) drop [31]. As a dopant to the liquid crystal matrix, other chemical objects such as dyes can also be also used [32].

Moreover, it is well-known that when reducing the size of the dopant material to the nanometric scale, the physical and chemical properties are changing [33], and in the case of nanoparticles, their properties strongly depend on their diameter [27].

Our goal was to obtain multifunctional material with advantageous electric, optic and magnetic properties. As is known, liquid crystal can be used as a medium for self-assembly of low concentrations of nanoparticles, due to its orientational and positional order [30]. In this paper, we present the influence of Fe_2_O_3_ nanoparticles with diameter below 50 nm on the physical properties of the liquid crystalline matrix. We have chosen Fe_2_O_3_ nanoparticles as an admixture because they have various unusual physical properties, are easy to synthesize and inexpensive, are non-toxic and have pronounced antibacterial properties; moreover, they are stable in an interesting temperature range (0–110 °C) [26,34,35,36]. Currently, the main obstacle to the use of the ferroelectric liquid crystal in display technology is the shock problem, i.e., irreversible destruction of an aligned LC layer by mechanical stress, although Pozhidaev et al. have recently reported on the synthesis of shock-free ferroelectric liquid crystal FLC-595 [37]. Nevertheless, we believe that the nanoparticles introduced into the ferroelectric liquid crystal matrix can also eliminate the shock problem. However, it requires a lot of studies using various types of nanoparticles with different concentrations. In this paper, we particularly focused on the effect of the admixture on the ferroelectric SmC* phase, and it was found that many of the LC matrix properties were changed after doping (in the synthesized soft magnetoelectric composites).

## 2. Materials and Methods

### 2.1. Samples

**Figure 1:**
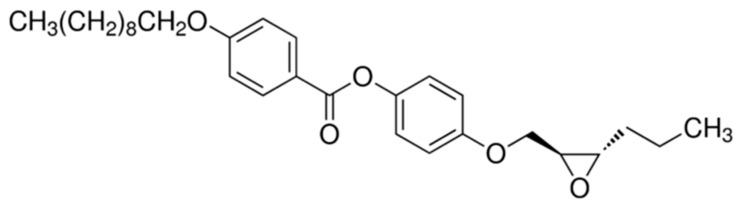
Molecular structure of the EHPDB matrix. The two chiral centers are on the carbons of the epoxy group.

As a ferroelectric liquid crystal matrix, we used a commercially available compound (short name EHPDB) (Sigma Aldrich Co., Saint Louis, MO, USA), belonging to the homologous series of epoxides, with the chemical formula presented in Figure 1. The epoxides are organic chemical compounds which contain a three-membered cyclic group consisting of an oxygen atom and two carbon atoms. The pure EHPDB compound was then doped with commercially available Fe_2_O_3_ nanoparticles (diameter < 50 nm, Sigma Aldrich Co., Saint Louis, MO, USA) to create six nanocomposites: Composites 1–5, with the concentrations of Fe_2_O_3_ nanoparticles equal to 0.0, 0.3, 0.5, 0.7 and 0.9 wt.%, respectively (Composite 1 is in fact the pure EHPDB matrix). The concentrations of nanoparticles used were very low so as not to alter the liquid crystal order and the director in the individual smectic layers. Moreover, at higher concentrations, the nanoparticles can interact strongly with each other, causing uncontrolled aggregation (typical for various types of nanoparticles) [31]. Therefore, it is important to pay special attention to disperse the nanoparticles well in the liquid crystal host. Composites 2–5 were prepared according to the same scheme:

1. The adequate amounts of pure EHPDB and Fe_2_O_3_ nanoparticles were weighed into separate vials (see Table 1).
2. 1.5 mL of chloroform was added to the weighed amounts of nanoparticles at room temperature, and the solution was sonicated in 50 s(on)/10 s(off) mode for 45 min (A = 30%, ice blanket).
3. The nanoparticle solution was sequentially poured into a vial with pure matrix EHPDB and chloroform was added to 2 mL, then the solution was left for 15 min to dissolve the EHPDB.
4. The solution (EHPDB + Fe_2_O_3_ + chloroform) was sonicated in 50 s(on)/10 s(off) mode for 30 min (A = 30%, ice blanket).
5. The solution (EHPDB + Fe_2_O_3_ + chloroform) was kept on the heating plate (80 °C) for about 150 min in order to obtain about 1 mL of solution.
6. The solution (EHPDB + Fe_2_O_3_ + chloroform) was sonicated in 50 s(on)/10 s(off) mode for 15 min (A = 30%, ice blanket).
7. The prepared solution was poured into microscopic slides and dried for at least 36 h at room temperature to evaporate the chloroform.
8. Obtained composites were peeled off with a blade and used for measurements.

Composite 1 (pure EHPDB) was prepared in the same was, but steps related to weighing and sonication of nanoparticles were omitted. Due to the use of very low concentrations of nanoparticles and the multi-stage sonication process, it was decided not to use a surfactant (e.g., oleic acid) in the composites. However, to check the influence of oleic acid on the composites’ parameters, we prepared Composite 6, where the concentration of nanoparticles was equal to 0.3 wt.% (like in Composite 2), but they were decorated with oleic acid before being put into the EHPDB matrix (see the Supplementary Materials). Additionally, we prepared two samples of Fe_2_O_3_ nanoparticles (see the Supplementary Materials) to check the difference between pure nanoparticles (Sample 1) and nanoparticles decorated with oleic acid (Sample 2). Mass compositions of Composites 1–6 and Samples 1 and 2 are presented in Table 1.

### 2.2. Powder X-ray Diffraction (PXRD)

Powder XRD diffractograms for samples studied were collected on a RIGAKU MiniFleX600 diffractometer (Rigaku, Tokyo, Japan) equipped with monochromatic Cu-Kα radiation (*λ* = 1.5406 Å). Powder samples were placed in zero-diffraction Si plates and diffraction patterns were collected over a 2 θ range of 5–90°, at a scanning speed of 10°/min. This method was also used to characterize the Fe_2_O_3_ nanoparticles and to check how small concentrations of nanoparticles modify the structure of the liquid crystalline matrix.

### 2.3. Optical Microscopy (OM)

Digital optical microscopy images have been collected at room temperature by using a Keyence VHX–7000 digital microscope (Keyence Corporation, Osaka, Japan). Images were obtained in transmittance mode at 80×, 400×, 2000× and 6000× magnifications, for samples manually placed on a microscope glass slide of 1 mm thickness. The high-resolution optical microscopy method was used to characterize the surface morphology and the distribution of nanoparticles in nanocomposites, and moreover to obtain preliminary information about the influence of oleic acid on the nanoparticles.

### 2.4. Scanning Electron Microscopy (SEM)

A Keyence VHX-D510 scanning electron microscope (Keyence Corporation, Osaka, Japan) was used to register the topography of the studied materials. Samples were mounted to the SEM specimen stubs with the double-sided adhesive copper tape without an additional sputter-coating with metal to preserve the original color and morphology of materials. Detailed imaging of all samples has been performed at 100×, 1000×, 5000× and 10,000× magnifications at room temperature.

### 2.5. Differential Scanning Calorimetry (DSC)

The calorimetric curves for Composites 1–6 were collected in the temperature range from 0.0 to 110.0 °C during heating and cooling at several chosen rates (5, 10, 12, 15, 20 °C/min) by using a PerkinElmer DSC8000 differential scanning calorimeter (Perkin Elmer, Waltham, MA, USA), calibrated on melting points of water and indium. The samples of exactly 5.13 mg were placed into aluminum pans and sealed by the press. Results were analyzed by using Pyris software (Perkin Elmer, Waltham, MA, USA) and Origin 2018 (OriginLab, Northampton, UK). Based on this method, the phase transition temperatures at so-called zero rate were determined by a linear fit to the dependence of the onset temperature versus the heating/cooling rate.

### 2.6. Polarizing Optical Microscopy (POM) and Electro-Optic Measurements (EOM)

A Nikon Eclipse LV100POL polarizing optical microscope (Nikon, Tokyo, Japan) equipped with the Fine Instruments WTMS-14C heating stage (liquid nitrogen as a coolant, temperature uncertainty of 0.1 °C) was used for texture observations. The experimental set-up including the above-mentioned microscope additionally consisted of the 33120A Agilent generator, the F20-FLC Electronics amplifier, the DSO6102A Agilent oscilloscope and the INSTEC PD02 photo-detector, and a system of tunable resistors (a 100 kΩ resistor was used in each case) was used for electric and electro-optic measurements. The digital camera Canon EOS600D (Canon, Tokyo, Japan) was used for texture acquisition during heating and cooling, with a rate equal to 2 °C/min, whereas the tilt angle and transmitted light intensities (TLI) measurements were performed by using a photo-detector. Composites 1–6 were placed by the capillary effect into sandwich-type ITO electro-optic cells (AWAT Company, Warsaw, Poland) (4.9 μm thick, 25 mm^2^ active electrodes surface) with the planar alignment [38,39,40], guaranteed by a thin polyimide layer (AWAT Company, Los Angeles, CA, USA). The uniformly aligned samples were obtained by heating to the isotropic phase and then slowly cooling down to the first tilted smectic phase, SmC*, where an external electric field was applied for a few minutes to align the samples and obtain the monodomain. The reversal current method was used to measure spontaneous polarization [41,42], tilt angle was measured as half of the angle between the Clark and Lagerwall states [43], while the switching time was measured as the time delay in the sample response [42,44]. An alternating voltage of 60 V_pp_ was used to measure spontaneous polarization, switching time and tilt angle. The surface area determination in the sample response by numerical integration is subject to uncertainty at the level of 2%, while the tilt angles for the off states are determined with a systematic uncertainty equal to 0.1°. All raw data were collected by specially prepared software, while the analysis was performed using Origin 2018.

The helix pitch of Composites 1–5 was measured for free droplets by using the BX–53F Olympus microscope (Olympus, Tokyo, Japan) equipped with the Instec ST402 hot stage and mK1000 temperature stabilizer. The images were registered during very slow cooling (0.1 °C/min) from the isotropic phase, after a one-minute isothermal time before each measurement to produce strips corresponding to the helix length. The phase transition temperatures in this experiment are shifted to higher temperatures due to the nature of the measurement (free droplet placed on the microscope slide is heated only from the bottom). Due to the large helix pitch, it was not possible to use UV-Vis spectroscopy [45].

Transmitted light intensity (TLI) through the sample placed in the ITO cell was measured to find the transition temperature. A standard measuring set as for electric measurements with a photo-detector was used (INSTEC, Boulder, CO, USA).

### 2.7. Fluorescent Confocal Polarizing Microscopy (FCPM)

The confocal microscopy is a recognized technique of three-dimensional imaging, long used in biological and medical sciences, and recently also for the soft matter research [46,47]. In our studies, we employed the Olympus FLUOVIEW FV1000 IX81 confocal microscope (Olympus, Tokyo, Japan) in the reflection mode with a diode laser (λ_D_ = 473 nm, beam power = 1.24 mW) [48]. To obtain a 3D image of a sample, a focused beam scans the sample in a horizontal plane (x, y directions) at different depths of the sample (z direction). Before measurement, each sample was treated with chloroform and then poured onto a microscope slide. After the evaporation of chloroform, the slide was subjected to the microscopic imaging.

### 2.8. FT-MIR (Fourier Transform-Middle Infrared Spectroscopy)

Fourier transform-middle infrared absorption measurements were performed in transmission mode using the Bruker VERTEX 70v vacuum spectrometer (Bruker, Billerica, MA, USA). The spectra were carried out in the range of 4000–450 cm^−1^ with spectral resolution of 2 cm^−1^ and 32 scans per each spectrum. All spectra processing was performed using Bruker OPUS software (Version 7.0, Bruker, Billerica, MA, USA). All samples were mixed with KBr, compressed into pellets and measured in two subsequent steps: first during heating from 20 up to 100 °C, and then cooling from 100 down to 10 °C. Additionally, pure oleic acid has been measured in the same measurement sequence for comparison. As the sample is a liquid, it was spread between two KBr pellets before measurement.

EHPDB (FTIR): 3113, 3102, 3070 ν(CH)_ar_; 2957, 2933; 2920s ν_as_(CH_2_)_al_; 2873; 2850m ν_s_(CH_2_)_al_; 1724s ν(C=O), 1730sh; 1607s, 1582, 1510s ν(C=C)_ar_; 1472, 1466m δ_as_(CH_2_)_al_; 1451m, 1420w ν(C=C)_ar_; 1392w, 1379w δ_s_(CH_3_)_al_; 1360w; 1342sh; 1313; 1300sh, 1288, 1278sh, 1257s, 1247sh ν_as_((C_ar_–O–C); 1200s ν_as_((C=O)–C), 1170s ν_s_((C=O)–C); 1106w, 1087s, 1080sh ν(C–O); 1064, 1051w ν_s_((C_ar_–O–C); 1025sh, 1019s, 1010s δ_ip_(CH)_ar_; 996sh, 971w; 902m ν(C–O)_epoxy_, 879m, 865; 850m δ_oop_(CH)_ar_; 829, 803m, 780m, 762s; 752sh, 741w; 725sh, 721m ρ(CH_2_)_n_; 694m, 653, 632, 594m, 536sh, 527m δ_oop_(ring)_ar_ cm^−1^ (where, s—strong, m—medium, w—weak, br—broad, sh—shoulder, ρ—rocking, ip—in plane, oop—out of plane, ar—aromatic, al—aliphatic, epoxy—in epoxy group, n—chain, S_s_—symmetric, as—asymmetric).

## 3. Results and Discussion

### 3.1. PXRD Results

**Figure 2:**
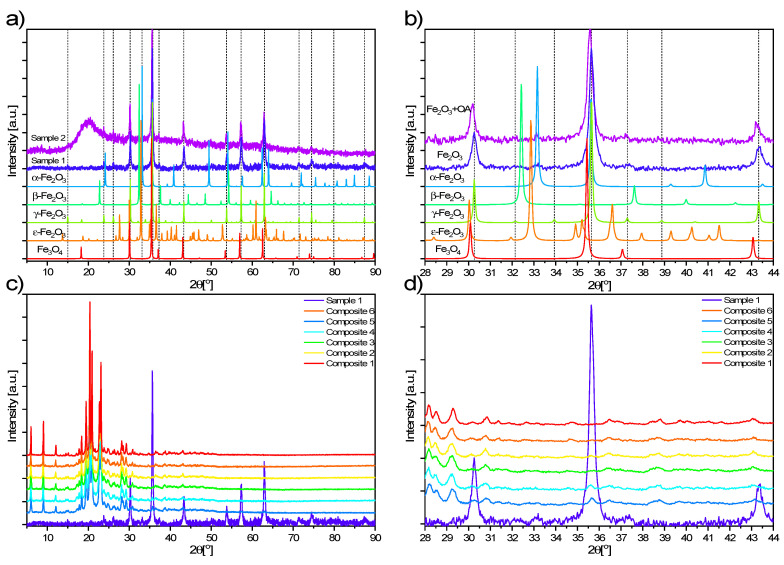
(**a**) XRD patterns of Sample 1 (Fe_2_O_3_), Sample 2 (Fe_2_O_3_ + OA) and reference materials in the ranges 5–90° and (**b**) 28–44°. (**c**) Dashed lines correspond to the positions of diffraction peaks for maghemite. XRD diffractograms of Composites 1–6 and Sample 1 registered in the ranges 5–90° and (**d**) 28–44°.

Figure 2a,b show the diffractograms of Sample 1 (Fe_2_O_3_) and Sample 2 (Fe_2_O_3_ + OA) as well as reference materials (hematite α-Fe_2_O_3_, metastable β-Fe_2_O_3_, maghemite γ-Fe_2_O_3_, Luogufengite ε-Fe_2_O_3_ and magnetite Fe_3_O_4_) [49,50,51,52,53,54,55,56,57,58]. The analysis of these data led to the conclusion that the Fe_2_O_3_ nanoparticles used in the syntheses of Composites 2–6 consist mainly of maghemite (γ-Fe_2_O_3_), however, very small impurities of hematite (α-Fe_2_O_3_) and magnetite (Fe_3_O_4_), by-products in the preparation of maghemite, may occur. Moreover, the broad amorphous peak of oleic acid with a maximum around 20° is recognized in the Fe_2_O_3_ + OA diffractogram.

Diffraction patterns of Composites 1–6 and Sample 1 are shown in Figure 2c,d. For all hybrid materials containing EHPDB liquid crystal matrix and Fe_2_O_3_ nanoparticles (Composites 2–6), the diffraction patterns are similar to pure EHPDB (Composite 1), supplemented with low-intensity peaks characteristic of maghemite, the intensity of which increases with increasing Fe_2_O_3_ concentration, confirming the presence of both components in these investigated samples.

### 3.2. OM Results

**Figure 3:**
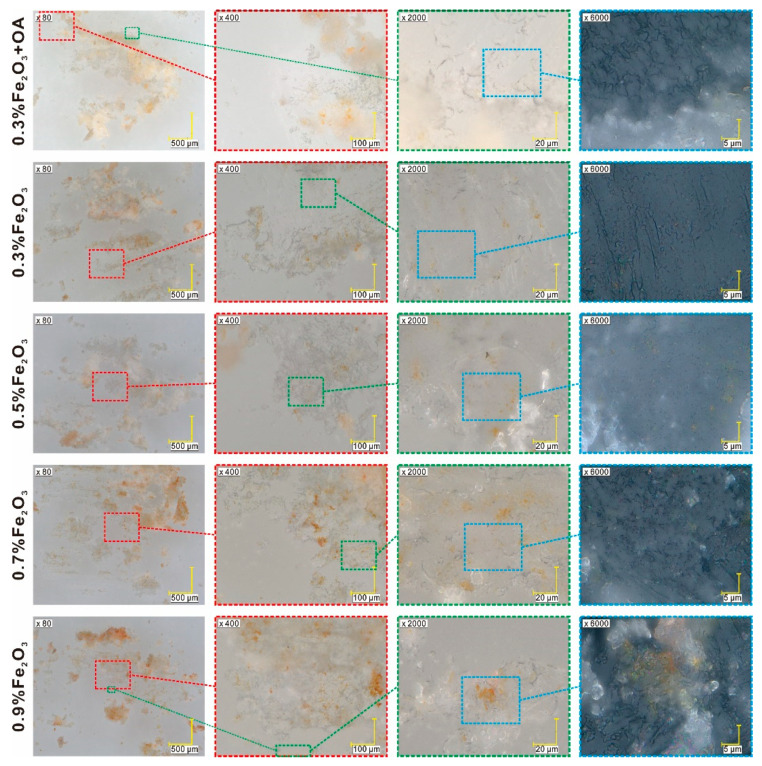
Digital optical images of Composites 1–5. The magnification is shown in the images, where colored dotted squares correspond to the enlarged areas.

The Fe_2_O_3_ nanoparticles (Sample 1) adopted the form of brown/orange powder, consisting of the agglomerates of maghemite (γ-Fe_2_O_3_) nanoparticles with a size of approximately 100 nm, which can be recognized as black dots in the image at 6000× magnification (Supplementary Figure S1). In turn, Sample 2 (Fe_2_O_3_ + OA) is much more homogenous without the large agglomerates of nanoparticles, while Composite 1 (pure EHPDB) forms colorless/white conglomerates of crystallites without any characteristic morphological features (see Supplementary Figure S1). Images for Composites 2–5 are similar (Figure 3), showing yellowish-white areas with orange inclusions of various sizes (corresponding to nanoparticle agglomerates), the color intensity of which is proportional to the concentration of nanoparticles. The agglomerates (orange islands) are quite clearly visible for Composites 4 and 5, especially at 400× and 2000× magnification, while they are not observed for nanocomposites with lower concentrations of nanoparticles (Composites 2 and 3, see Figure 3). It is worth emphasizing that in the sample fragments that appear uniform, single, well-dispersed Fe_2_O_3_ nanoparticles are visible in the form of small dark dots in images with a magnification of 6000 times (Figure 3).

### 3.3. SEM Results

The SEM imaging of Sample 1 (Fe_2_O_3_ nanoparticles) revealed that the sample contained nanoparticle aggregates smaller than 100 nm in size (Supplementary Figure S2, first row). The addition of oleic acid to nanoparticles (Sample 2) makes the surface smoother, which proves good dispersion of nanoparticles and no agglomeration. Analysis of Composite 6 (0.3 wt.% Fe_2_O_3_ + OA) images showed that the sample surface is smoother than Composites 2–5, with few ripples on the surface. Morphologies of Composites 2–5 are very similar, and the domains of the EHPDB matrix are clearly visible, however, these domains are not visibly modified by the dopant material even at 10,000× magnification (Supplementary Figure S2). The highest magnification of the microscope used is 10,000×, so we were not able to visualize individual nanoparticles and determine their exact size distribution, or even observe smaller aggregates. However, based on the images obtained, we can confirm that oleic acid prevents aggregation.

### 3.4. FCPM Results

**Figure 4:**
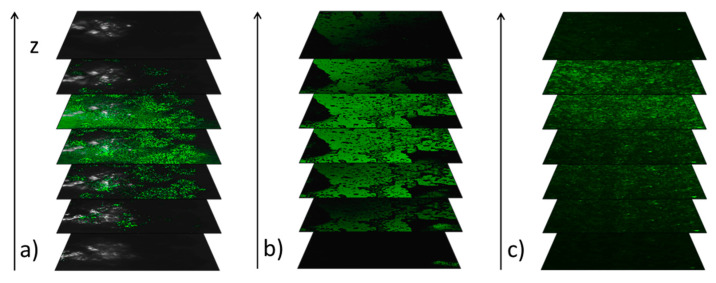
The qualitative representation of the fluorescence intensity in the subsequent slices of Sample 1 (**a**), oleic acid (**b**) and Composite 6 (**c**). The z axis is perpendicular to the x–y plane (parallel to the surface of the microscope slide).

As we have shown above, the use of a surfactant (oleic acid) to decorate nanoparticles aims to eliminate the aggregation process. However, when the matrix is doped with a low concentration of nanoparticles, the surfactant is an additional chemical agent that can act on both the organic matrix and the nanoparticles. In our opinion, for such small nanoparticle concentrations in Composites 2–6, the surfactant is a parasitic factor, and its use can complicate/obscure the matrix–nanoparticle interaction, and the modification of physicochemical parameters of composites may result from the use of the surfactant itself, not nanoparticles. Due to the lack of a coherent and complete description of the influence of surfactants in organometallic composites, we decided to check the effect of oleic acid on the aggregation process and the change in the parameters of the organic matrix in such composites. For this purpose, we used fluorescence imaging of thin films by confocal microscopy. Figure 4 presents the fluorescence of the subsequent slices of the investigated materials.

As one can see, both Fe_2_O_3_ nanoparticles and oleic acid exhibit the fluorescence, while the EHPDB matrix does not. Images of Sample 1 (Fe_2_O_3_ nanoparticles) present strong centers of fluorescence (intense green spots), which clearly proves the agglomeration, while images of oleic acid show uniform fluorescence over the entire surface and thickness (Figure 4a,b). On the other hand, the images recorded for Composite 6 (Figure 4c) show an intense and multi-site fluorescent signal, which in our opinion, for such a low concentration of nanoparticles (0.3 wt.%), comes mostly from oleic acid, not nanoparticles. However, these images further prove that the nanoparticles are quite well-dispersed in the EHPDB matrix.

### 3.5. DSC Results

**Figure 5:**
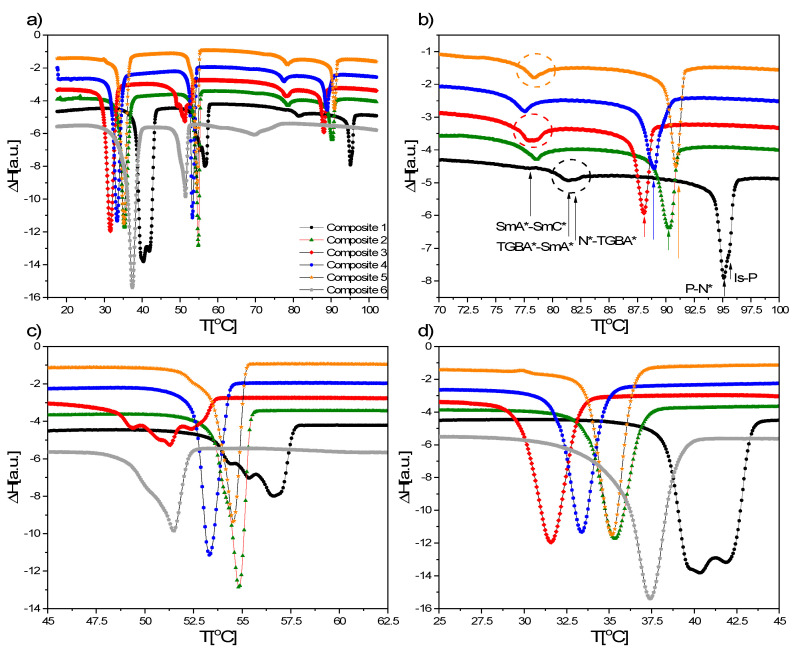
(**a**) DSC curves registered during cooling at a rate of 10 °C/min for Composites 1–6 and (**b**) extended temperature ranges of 100.0–70.0 °C, (**c**) 62.5–45.0 °C and (**d**) 45.0–25.0 °C.

As an example, the DSC curves registered during cooling (10 °C/min) for Composites 1–6 are presented in Figure 5a, and as it is clearly seen, the main differences in the phase transitions are for Composite 6 (with oleic acid). Taking into account the transition from isotropic liquid to N* (Figure 5b), it can be seen that for Composite 1 (pure EHPDB), two close anomalies can be distinguished, corresponding to two phase transitions (two black arrows). We suppose that the blue phase (BP) can exist in a very narrow range (marked by P in Figure 5b), although the characteristic texture of this phase was not observed under a polarizing microscope. It is well-known that blue phases are thermodynamically distinct phases that appear between the helical phase and the isotropic liquid in highly chiral liquid crystals. The EHPDB compound fulfils both conditions—it exhibits an isotropic liquid—N* phase transition and is a highly chiral compound. For Composites 2 and 4, a subtle anomaly indicates that this BP phase is also present, while for Composites 3 and 5, it is not. However, this is not sufficient evidence for the presence of BP in the studied composites, so it requires further research, which was not the aim of our study. After addition of nanoparticles to the liquid crystal matrix, the Is–N* phase transition temperature is shifted to lower temperatures (Figure 5b), and the smallest shift is for Composite 5 (0.9 wt.%), which may suggest that for concentrations greater than 0.9 wt.%, the shift will be even smaller, which in turn may indicate the complete nanoparticles’ agglomeration and even degradation of liquid crystalline properties. The N* phase for all Composites occurs in a relatively wide temperature range, which was also confirmed by texture observations. In turn, the temperature of the N*–SmA* transition is decreasing with increasing the concentration of nanoparticles to 0.7 wt.% in the Composites. Interestingly, for Composites 1, 3 and 5, in the vicinity of this phase transition, a second weak anomaly can be seen, while for Composites 2 and 4, it cannot (Figure 5b). We suppose that an additional very narrow frustrated smectic A* phase, namely TGBA*, appears [59]. The SmA*–SmC* phase transition is strongly shifted to lower temperatures for Composites 2–5, thus doping broadens the SmA* phase but narrows the SmC* phase range. The drastic narrowing of the SmC* phase due to such small nanoparticle concentrations is not fully understood and requires further research. It is not excluded due to the nanoparticles’ mobility in the liquid crystal matrix, and the orthogonal and positional order of the SmA* phase is maintained over the bigger temperature range, which causes the extension of the SmA* phase. At lower temperatures, the viscosity of the liquid crystalline phase increases, movement of nanoparticles is hindered and the transition to the ferroelectric phase begins. In the case of SmC*–Cr transition, the nanoparticles modify not only the onset and peak temperature, but also cause the disappearance of the polymorphism in the range of 57.5–47.5 °C (for Composite 1, the ferroelectric phase changes into a sequence of crystalline phases). Although it is not fully understood and it cannot be explained in a simple way, this proves that nanoparticles in low concentrations can cause the disappearance of not only the liquid crystalline but also the crystal phases (see Figure 5c,d). According to Yadav et al., aggregated nanoparticles act as impurities and decrease the temperature in nanocomposites [24].

As it was mentioned above, oleic acid strongly affects the mesophases (only the N* phase exists for Composite 6 during heating) as well as modifies the phase transition temperature—a very strong shift of the Is–N* phase transition to low temperatures and the narrowing of the mesophases (N* and SmC*) are visible (Figure 5a). On the other hand, the transitions within the crystal phase are not modified much by oleic acid. In the case of Composite 6 (with oleic acid), the Is–N* transition is shoulder-shaped, while the N*–SmA* transition is already visible as a complete anomaly (as in Composition 2 without oleic acid). Under the polarizing microscope, we did not observe uniform transitions as for Composites 1–5, but a drop in formation of the individual phases (only the transition to the crystal covers the entire visible surface). Probably due to the excessive amount of oleic acid used to decorate nanoparticles, it contributes to the destruction of the liquid crystalline properties. Due to the significant influence of oleic acid on the phase sequence of Composite 6, some studies (e.g., electrical, dielectric) were omitted. Interestingly, Da Cruz et al., by doping 5CB with γ-Fe_2_O_3_, also obtained nanocomposites with strongly reduced N–Iso phase transition temperatures, but finally found that it was caused by a surfactant, not nanoparticles [60]. In our case, we did not use a surfactant for Composites 2–5, so we cannot attribute the temperature shift to anything other than nanoparticles.

During heating, the registered curves were similar for Composites 1–5, however, again, a shift in the phase transition temperatures (particularly in the N*–isotropic liquid phase transition) was observed (see Supplementary Figure S3). A shift of the phase transition temperatures for hybrid materials based on 11OBA and 12OBA liquid crystals doped with Fe_3_O_4_ nanoparticles (1–3 wt.%) was also observed by Sivasri et al. [61]. In turn, for Composite 6 (with oleic acid), only two anomalies were visible on the heating curve, which is further evidence of the degrading effect of this surfactant.

### 3.6. TLI Results

**Figure 6:**
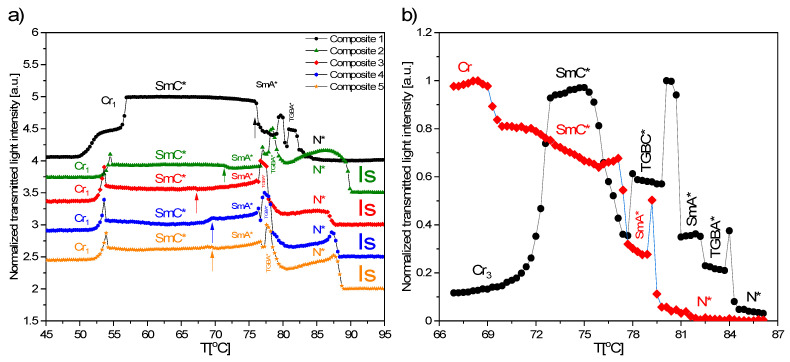
Normalized transmitted light intensity registered during cooling (**a**) and heating (**b**), rate = 3 °C/min.

Some of the phase transition temperatures were not designated by the DSC method, therefore the complementary method, TLI, was used, mainly to determine the SmA*–SmC* phase transition temperature. This phase transition is strongly shifted to lower temperatures for all doped samples. Thus, doping with Fe_2_O_3_ nanoparticles widens the SmA* phase range but narrows the SmC* phase range. The normalized transmitted light intensity versus temperatures registered for all composites studied is presented in Figure 6, and as is seen, this transition is a very subtle small hump for Composites 2–5, while for Composite 1 (pure EHPDB), it is quite visible (arrows in Figure 6a). Interestingly, the presence of the TGBA* phase in all composites and an extra phase (probably the BP phase) in Composite 1 were confirmed. Intensity of transmitted light is almost constant within the SmC* for all composites, however it increases to a small extent for the pure matrix. This behavior can be connected with the defected texture of the ferroelectric SmC* phase for Composites 2–5, which was observed by polarizing imaging.

### 3.7. POM Results

**Figure 7:**
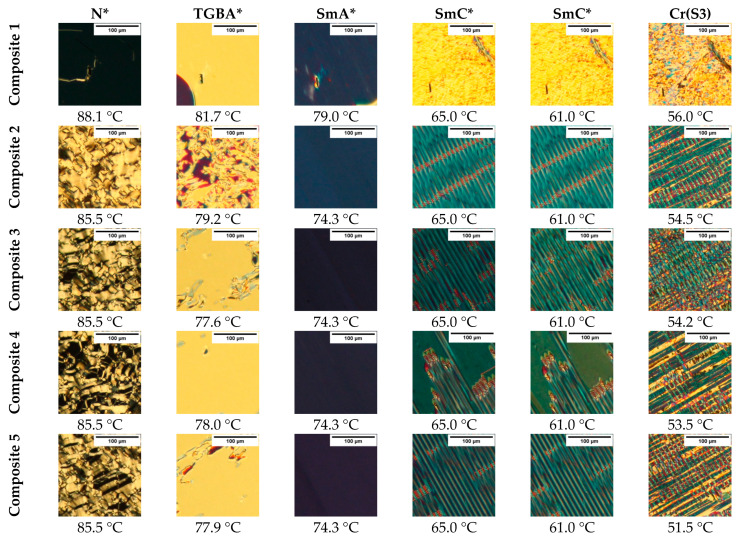
Textures of Composites 1–5 registered during cooling (2 °C/min) at the same light intensity. The last column represents the first observed crystalline phase. The textures within each composite represent the same area.

As an example, textures for Composites 1–5 registered for the planar ITO cells during cooling are presented in Figure 7. The admixture of Fe_2_O_3_ nanoparticles strongly influences the interference image of a thin layer, regardless of the concentration. In the case of N* phase, the oily-streaks texture was registered for Composite 1, while for Composites 2–5, the fan-shaped texture was registered (see Figure 7, first column). Basically, the nucleation of the N* phase is in the form of irregular droplets that aggregated very quickly to form a fan-like texture, and with further decreasing temperature, the fan-like texture began to crack and streaks were formed (see Supplementary Figure S4).

On further cooling, the frustrated TGBA* phase, as Grandjean texture (uniform in color) with red inclusions, occurred in a very narrow temperature range, and no significant influence of Fe_2_O_3_ nanoparticles on this texture was observed. A similar texture for the TGBA* phase was observed by Chakraborty et al. for the two-component mixture of 2H6R + H–22.5 [62], although usually, the N*–TGBA* phase transition is associated only with smudging the texture, e.g., oily-streaks, and it is very often difficult to detect [63]. In turn, the SmA* phase for all composites forms a homogeneous texture [63], while the strong influence of the nanoparticles on the texture of the ferroelectric SmC* phase is clearly visible. Composites 2–5 form a striped texture, not observed for Composite 1 (pure EHPDB) at any temperature. It is very likely that the striation induced by nanoparticles is a certain electrohydrodynamic instability that occurs in nanocomposites in the form of elongated domains (stripes) [64,65] or/and the formation of ferroelectric domains in nanocomposites [66]. However, this requires further investigation. Misra et al. doped the ferroelectric liquid crystal mixture SCE-13 with spherical Fe_2_O_3_ nanoparticles (ca. 6–12 nm, 1 wt.%) and observed the emergence of focal-conic domains [67]. Thus, it is not excluded that the size of the nanoparticles determines the texture-striated (larger nanoparticles) and focal-conical domains (smaller nanoparticles). However, this hypothesis requires further research. As the temperature decreases, the linear pattern disappears, and a crystal phase appears (Figure 7). However, in our previous study, we reported on the presence of a metastable S3 phase (unclassified smectic phase) immediately below the SmC* phase for pure EHPDB [68]. Thus, it is not excluded that the presented texture for the first crystal phase is that for the S3 smectic phase.

**Figure 8:**
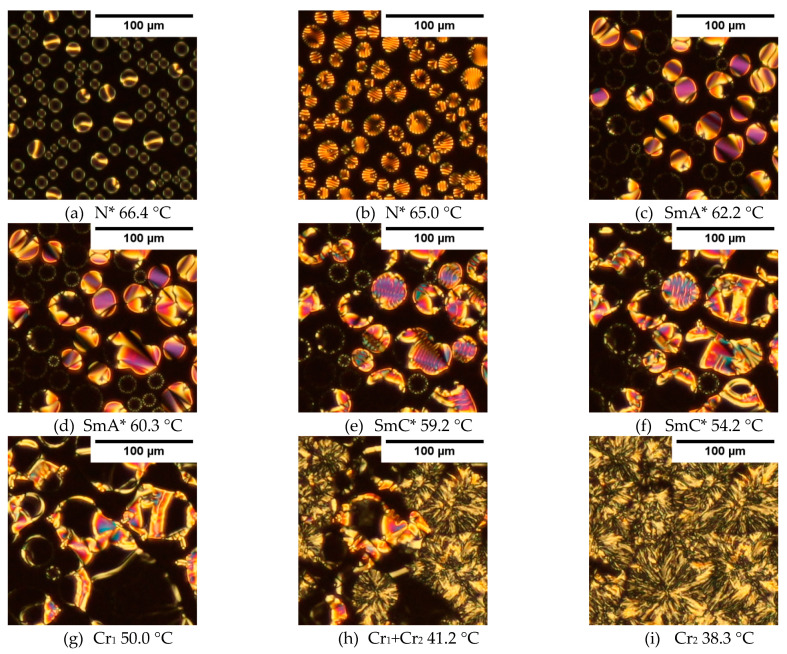
Textures of Composite 6 registered during cooling at a rate of 2 °C/min in the N* (**a**,**b**), SmA* (**c**,**d**), SmC* (**e**,**f**) and crystalline phases (**g**–**i**).

The textures registered during cooling for Composite 6 (with oleic acid) are presented in Figure 8, and it is seen that the addition of oleic acid significantly modified the textures (compared with Figure 7). During the transition from the isotropic liquid to the N* phase, many fine regular droplets of different diameters were visible (Figure 8a), which, on further cooling, coalesced into larger droplets and stripes appeared on them (Figure 8b). This behavior may indicate changes in the N* phase birefringence and helix pitch. In the SmA* phase (Figure 8c), some of the drops observed in the N* phase were visible as color droplets with focal-conic texture, while some of them as dark droplets with a visible border. It seems that droplets with a sufficiently large radius in the N* phase are also colored in the SmA* phase, while those with a radius below a certain threshold radius become dark (change the orientation of the molecules from planar to homeotropic). We suppose that in Composite 6 (with oleic acid), from the energetic point of view, the arrangement of molecules in the homeotropic configuration is more favorable, as it was explained by Zakerhamidi et al. that oleic acid (on the surface of nanoparticles) can orient the liquid crystal molecules to a homeotropic state due to the strong coupling between the oleic acid chains and the liquid crystal molecule [69]. However, in our case, the concentration of the nanoparticles was small (0.3 wt.%), but the dark area of the texture was large, and therefore it seems that part of the dark texture does not contain nanoparticles and liquid crystalline molecules (is not homeotropic), but rather oleic acid itself (excessive amount of oleic acid). In turn, if the boundaries are left of the small droplets, it may suggest that there is a homeotropic alignment inside the droplet, while at the edge, the molecules are tilted. As the SmA* phase is cooling down, the droplets became bigger (Figure 8d), and after transition to the SmC* phase (recognized by the characteristic fingerprint stripes), they continue to increase (Figure 8e,f). The striation in droplets disappeared with further cooling, and transition to a crystal phase was visible (Figure 8g) as droplets merged. During further cooling, the coexistence of two crystal phases was clearly visible (Figure 8h), while a low-temperature crystal phase is presented in Figure 8i. It is possible that Cr_1_ is an unclassified smectic phase (S3), as we mentioned above. XRD studies or the effect of a very strong electric field on the texture of the Cr_1_ phase could help identify this phase, but we were not able to perform such studies due to the lack of a sample. However, due to the interpretation difficulties, we consider this as a crystal phase. To sum up, on the one hand, OA inhibits the aggregation of nanoparticles in the LC matrix, but at the same time, it has a strong influence on the phase sequence, and therefore the remaining studies were conducted only for Composites 1–5 (without oleic acid).

**Figure 9:**
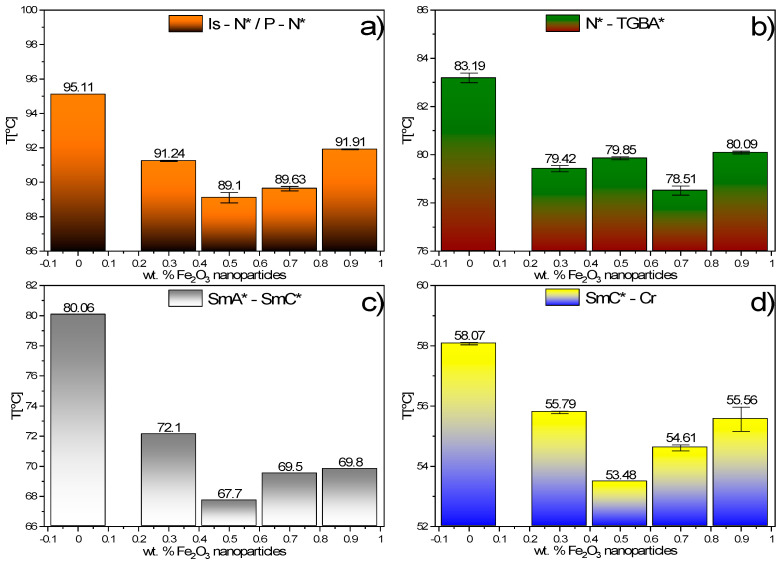
Influence of Fe_2_O_3_ nanoparticles admixture on the phase transitions during cooling: Is–N*/P–N* (**a**), N*–TGBA* (**b**), SmA*–SmC* (**c**) and SmC*–Cr (**d**).

Based on the results presented above (POM, DSC, TLI), the phase sequences for Composites 1–6 were determined (see Supplementary Table S1). Figure 9 presents the influence of the Fe_2_O_3_ nanoparticles on the phase transition during cooling, while Supplementary Figure S5 presents them during heating. Nanoparticles’ addition broadens the paralectric SmA* phase and narrows the ferroelectric SmC* one. We suppose that the mobile nanoparticles move quite freely in the organic matrix in the SmA* phase, which hinders the phase transition to the tilted phase. When the temperature decreases, the mobility of nanoparticles drops, and phase transition follows.

### 3.8. EOM Results

**Figure 10:**
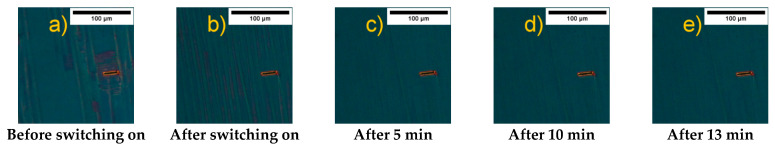
The alignment process of Composite 5 (20 V_pp_, 50 Hz, rectangular signal).

All electro-optic measurements were performed on the uniformly aligned samples. Figure 10 presents, as an example, the alignment process of Composite 5, while Supplementary Figure S6 of Composites 1–5. The ordering conditions were selected experimentally (the rectangular wave, 50 Hz). The textures of doped samples (Composite 2–5) before alignment are quite similar, but different than for pure EHPDB (Composite 1). After applying the electric field, the monodomain of good quality was immediately formed (Figure 10 and Supplementary Figure S6), and after ca. 13 min, the uniformly aligned samples (in the whole area of the electrodes) were obtained. The very good quality of the monodomain proves that the helical pitch is infinite [63].

**Figure 11:**
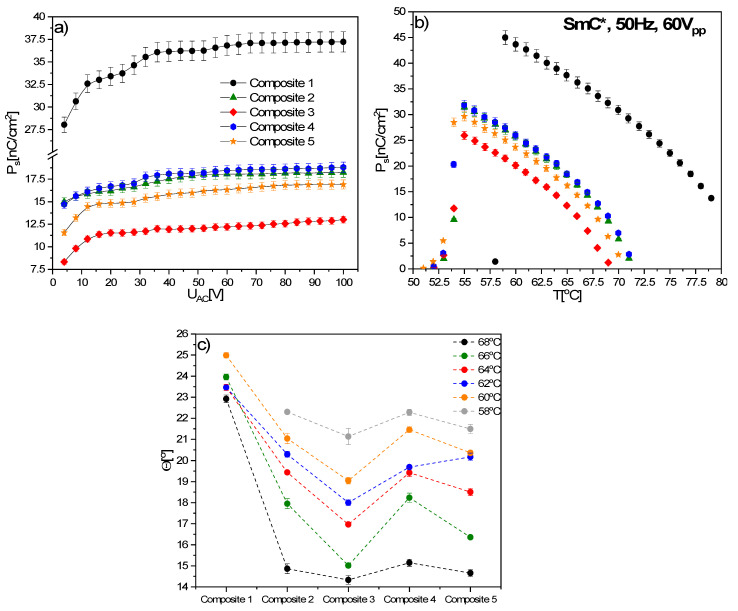
Spontaneous polarization versus applied voltage (50 Hz, triangular wave) in the SmC* phase (65.0 °C) for Composites 1–5 (**a**), temperature dependence of spontaneous polarization, the accuracy of the P_s_ is 3% (**b**), and tilt angle of Composites 1–5 at several selected temperatures (**c**). Dashed lines are a guide for the eye.

As can be seen in Figure 11a, a non-zero polarization was observed for each sample already at a voltage of 4.0 V (the voltage threshold associated with the reorientation of molecules is below 4.0 V), while the saturation was observed at different voltages, depending on the Composite. From about 30.0 V, a very slight increase in spontaneous polarization was observed for all Composites. For Composite 1 (pure EHPDB), the spontaneous polarization was saturated at 60.0 V, for Composites 2 and 4, at around 80.0 V, while for Composites 3 and 5, after exceeding 30.0 V, a continuous slow increase in spontaneous polarization was visible. The spontaneous polarization at 100.0 V is equal to 37.22, 18.25, 13.01, 18.82 and 16.90 nC/cm^2^ for Composites 1–5, respectively. It turned out that even the smallest amounts of nanoparticles caused a drastic decrease in spontaneous polarization. Interestingly, there is no close relationship between the concentration of Fe_2_O_3_ admixture and the decrease in P_s_ values. For the nanoparticle content of 0.7 and 0.3 wt.%, a two-fold decrease, while for 0.5 wt.%, an almost tripled decrease, in P_s_ were observed. In turn, for the content of 0.9 wt.%, the P_s_ was higher than for 0.5 wt.%. Decreasing of the threshold voltage for the reorientation of LC molecules was also obtained with the use of other types of nanoparticles, such as BaTiO_3_ and Sn_2_P_2_S_6_ for the compounds Merck 18523 and LC 1550 [70]. In turn, an increase in the threshold voltage after doping with graphene flakes was observed, which was explained by anchoring molecules to the surface of graphene flakes [71].

Temperature dependence of the spontaneous polarization for Composites 1–5 is presented in Figure 11b. Since the SmA*–SmC* phase transition is of the second order, continuous growth of the spontaneous polarization was observed for Composites 2–6. On the other hand, for Composite 1 (pure EHPDB), at the transition temperature (ca. 80 °C), a sudden jump was visible, which is related to the difficulties of measuring the phase transition. The spontaneous polarization does not saturate for any sample, while it decreases after doping, and the maximum value of P_s_ is equal to 44.99, 31.45, 25.94, 31.85 and 29.67 nC/cm^2^ for Composites 1–5, respectively.

Novotná et al. doped liquid crystal with SmC* phase with Fe_2_O_3_ nanoparticles (ca. 4 nm) decorated with oleic acid, and also observed a decrease in the spontaneous polarization with increasing the dopant concentration [30]. The same behavior was observed by Khushboo et al. for the ferroelectric W206E mixture doped with NiFe_2_O_4_ nanoparticles (ca. 45 nm, 0.12, 0.25, 0.50 wt.%) [72]. In turn, Prodanov et al. doped a ferroelectric liquid crystal mixture with CoFe_2_O_4_ nanoparticles (ca. 7.8 nm, 1 and 3 wt.%) and also observed a decrease in P_s_ at lower concentrations, and practically no change at a higher dopant concentration, e.g., similar to the studied composites [73]. Interestingly, Popova et al. doped a ferroelectric liquid crystal with γ-Fe_2_O_3_ nanoparticles (ca. 15 nm, 0.01–0.02 wt.%) and observed no dopant influences on the spontaneous polarization [74], probably due to the low concentration of the admixture. Therefore, it can be concluded that the admixture of Fe_2_O_3_ nanoparticles usually reduces the spontaneous polarization. However, it is also not excluded that the spontaneous polarization decreases due to the electrode polarization phenomenon.

As an example, the tilt angle for several selected temperatures in the SmC* phase for Composites 1–5 is presented in Figure 11c. As it is clearly shown, the tilt angle decreases in the same way, with a change of nanoparticle concentration in nanocomposites (Composites 2–5), as spontaneous polarization, and at 68 °C, it is equal to: 22.92°, 14.86°, 14.33°, 15.15° and 14.66° for Composites 1–5, respectively. The greatest decrease, about 8.6°, was found for Composite 3 (0.5 wt.%). Additionally, since the SmA*–SmC* transition temperature shifted to about 71 °C in Composites 2–5, it seems that Fe_2_O_3_ nanoparticles are responsible for the lack of molecular tilt above this temperature. We suppose that the decrease in the tilt angle and spontaneous polarization may be caused by the physical adsorption of organic molecules on the surface of nanoparticles and the strong interaction between nanoparticles in nanocomposites (hindering the reorientation of organic molecules).

In the sample response, during P_s_ measurements, two anomalies appeared: one related to the reorientation of organic molecules and the second probably related to the ions present in Composites 1–5 (coming from the synthesis). However, this second anomaly splits into two anomalies for Composites 2–5 (but not for pure EHPDB) when increasing the applied voltage. In our opinion, this third anomaly is related to the introduction of additional ions into the matrix along with the nanoparticles (chemically, other ions than those present in the pure matrix) or to the contamination of Fe_2_O_3_ nanoparticles with FeO, having a non-zero dipole moment [75]. Another possibility is that a dipole moment is induced in Fe_3_O_4_ nanoparticles (existing as impurities, see PXRD results) under an external electric field, as was mentioned by Zakerhamidi et al. [69].

**Figure 12:**
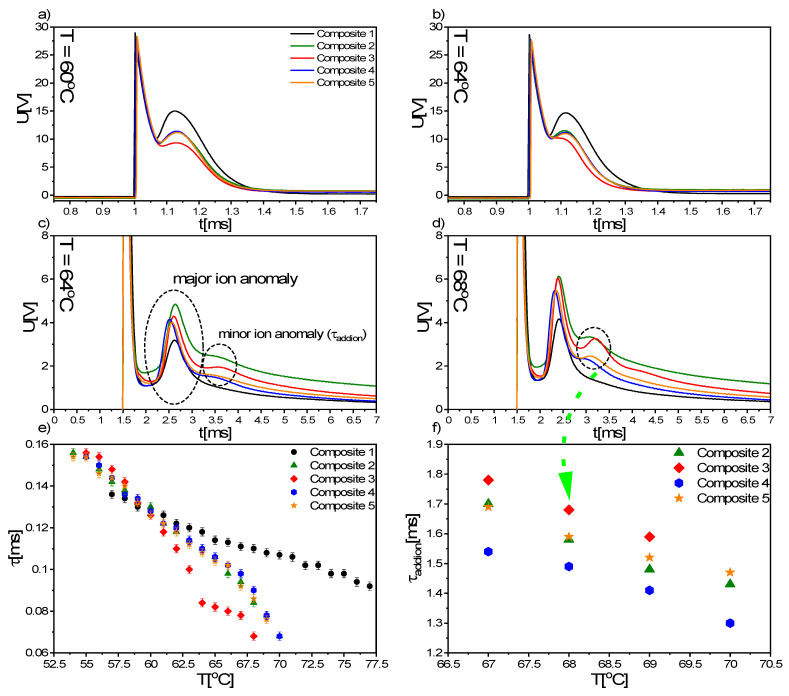
The voltage response of Composites 1–5 on rectangular wave (50 Hz, 20 V_pp_) at temperatures of 60 °C (**a**) and 64 °C (**b**), and on rectangular wave (50 Hz, 60 V_pp_) at 64 °C (**c**) and 68 °C (**d**). Temperature dependence of the switching time for Composites 1–5 (**e**) and reorientation time of additional ions at several selected temperatures for Composites 2–5 (**f**). The legend in graph (**a**) is the same for (**a**–**d**) graphs.

The switching time was determined from the sample response to the applied rectangular wave of 20 V_pp_, and for both the pure matrix and nanocomposites, was much shorter than a millisecond (Figure 12a,b). The ionic response visible for Composites 1–5, after increasing the applied voltage up to 60 V_pp_, split into two anomalies for nanocomposites: major and minor ionic anomalies, while for Composite 1, it did not (Figure 12c,d), as observed for spontaneous polarization. The reorientation time associated with the minor ion anomaly (τ_addion_) was determined for several selected temperatures, and it increased with the decreasing temperature for each nanocomposite (Figure 12f). Similarly, the switching time of organic molecules increased with decreasing temperature (Figure 12e), but this increase was much faster for nanocomposites than for the pure matrix. However, at low temperature ranges of the SmC* phase (below 58 °C), a shorter switching time was found for Composite 1 (pure EHPDB) than for nanocomposites (Composites 2–5).

**Figure 13:**
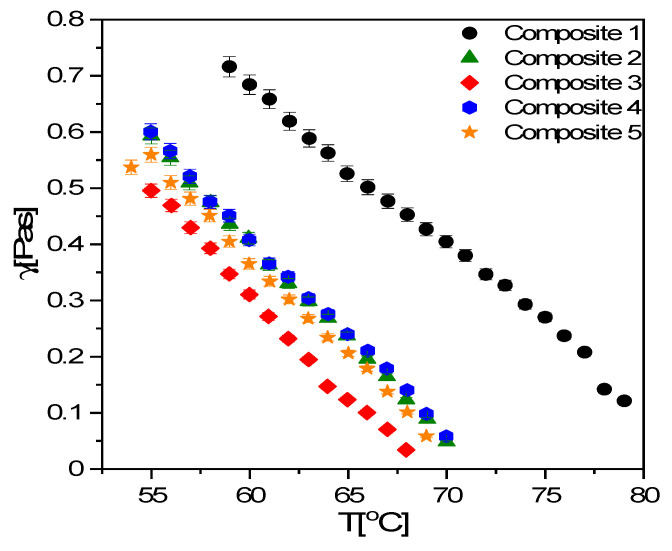
Temperature dependence of the rotational viscosity for Composites 1–5.

Based on the switching time and spontaneous polarization measurements, the rotational viscosity was determined (Equation (1)), and the results are presented in Figure 13.

(1) $$\gamma \left(T\right)=\tau \left(T\right)P_{s}\left(T\right)E_{applied}=\tau \left(T\right)P_{s}\left(T\right)\frac{U_{applied}}{d}$$

where *γ*, *τ*, *P_s_*, *E_applied_*, *U_applied_* and *d* are the rotational viscosity, switching time, spontaneous polarization, the electric field, applied AC voltage and sample thickness, respectively. As expected, the rotational viscosity increased with decreasing temperature for Composites 1–5, but due to the decrease of spontaneous polarization and shorter switching time, the rotational viscosity, *γ,* drastically decreased after doping (Figure 13). *γ* values are the same for Composites 2 and 4 (0.3 and 0.7 wt.%, respectively), but for Composites 3 and 5 (0.5 and 0.9 wt.%, respectively), they are smaller, within the measurement uncertainty. Although Goel et al. explained the decrease in the rotational viscosity in studied composites (Ni ca. 34 nm, KCFLC7S matrix) by nanoparticles’ adsorption to the ITO surface and modification of anchoring energy of molecules [76], we do not fully understand this behavior and have made an attempt to explain this. We suppose that the aggregation of nanoparticles is either absent or relatively small up to a concentration of 0.5 wt.% (Composite 3), but the agglomeration process becomes important for a higher concentration (0.7 wt.%, Composite 4), and it is even more important in Composite 5 (0.9 wt.%). In addition, Composite 5 can contain significantly more non-agglomerated nanoparticles than Composite 4, which further decreases spontaneous polarization and rotational viscosity.

The area of electrodes in the ITO cell used is 5 × 5 mm^2^. The width and the length of the LC molecule are ca. 5 Å and 40 Å [77] respectively, while the thickness of the smectic layer is about 37 Å (in tilted SmC* phase, tilt angle of 20°). Then, the electrode surface will cover about (5 mm/5 Å) × (5 mm/37 Å) = 1.35 × 10^13^ LC molecules, while about (4.9 μm/5 Å) × 1.35 × 10^13^ = 1.323 × 10^17^ LC molecules will be present in the volume of the electrodes (4.9 μm—thickness of the ITO planar cell). As it is known, the nanoparticle used is about 12.5 times larger than the liquid crystal molecule (the length of LC molecules L ≈ 40 Å, the nanoparticle size d ≈ 50 nm, d/L ≈ 12.5). Therefore, one nanoparticle occupies the volume of 14 LC molecules in one dimension in a single smectic layer of tilted phase (SmC*, θ ≈ 20°), and 14 × 14 × 14 = 2744 LC molecules in three dimensions. We used a very low concentration of nanoparticles for the synthesis of nanocomposites. If 1.323 × 10^17^ LC molecules will be present in the volume of the electrodes for pure EHPDB, 1.319 × 10^17^ LC molecules and 9.628 × 10^9^ nanoparticles will be present for Composite 2 (0.3 wt.%). Thus, the admixture of nanoparticles at such a low concentration eliminates a negligibly small amount of LC molecules and could not be responsible for the significant decrease of spontaneous polarization, tilt angle and rotational viscosity (if the P_s_ of Composite 3 is practically half of pure EHPDB, the introduced nanoparticles should eliminate half of the LC molecules). The FTIR results do not show that there is a chemical bond between nanoparticles and LC molecules (see below), however strong physical adsorption of molecules to the surface of nanoparticles cannot be ruled out. Besides, in the vicinity of the nanoparticle, the packing of LC molecules can be completely different than in the volume in which there are no nanoparticles. 

**Figure 14:**
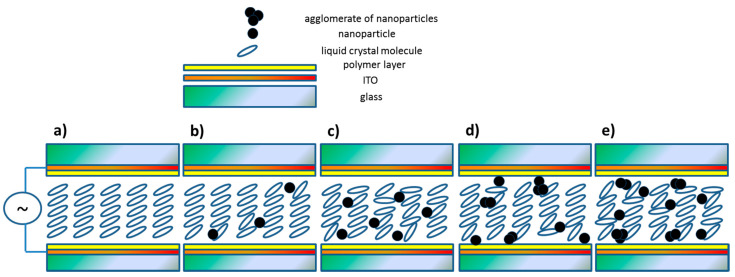
A schematic explanation of the spontaneous polarization behavior in Composites 1–5 with an increase in the concentration of Fe_2_O_3_ nanoparticles (**a**–**e**). Apart from the increase in the amounts of agglomerates in Composite 5 relative to Composite 4, the amount of non-agglomerated nanoparticles increases, which causes a further reduction in spontaneous polarization. The figures are not to scale, in fact the nanoparticles are much larger than the organic molecules. Ions present in the samples, which will reach the properly polarized electrodes under the influence of the AC field, were not taken into account.

Figure 14 schematically shows the effect of increasing the concentration of nanoparticles on the arrangement of molecules in a thin layer of composites in measuring the ITO cell. It should be stressed that the drawing is not to scale, i.e., the individual nanoparticle is larger than the liquid crystal molecule. In turn, Vimal et al., by doping ferroelectric liquid crystal Felix 17/100 with ferromagnetic nanoparticles (ZnO:Cu^2+^ ions, 1 wt.%), obtained the enhanced spontaneous polarization due to magneto-electric coupling [78]. To summarize, to explain the decrease in electrical parameters, further research is needed, for example, the SQUID method could provide an answer to the question of whether the interaction between nanoparticles (magnetic coupling) may disturb the switching process and cause the spontaneous polarization and the tilt angle of the molecules to decrease. Additionally, the interaction between two types of dipoles (electric for the LC molecules and magnetic for nanoparticles) can strongly modify the electric parameters of nanocomposites, and although the nature of this coupling is not fully understood, its existence cannot be ruled out [79].

Apart from studying the modification by doping the parameters typical for ferroelectric liquid crystals (spontaneous polarization, tilt angle, switching time), the anchoring energy coefficient of molecules to the cell surface and the helical pitch were determined.

**Figure 15:**
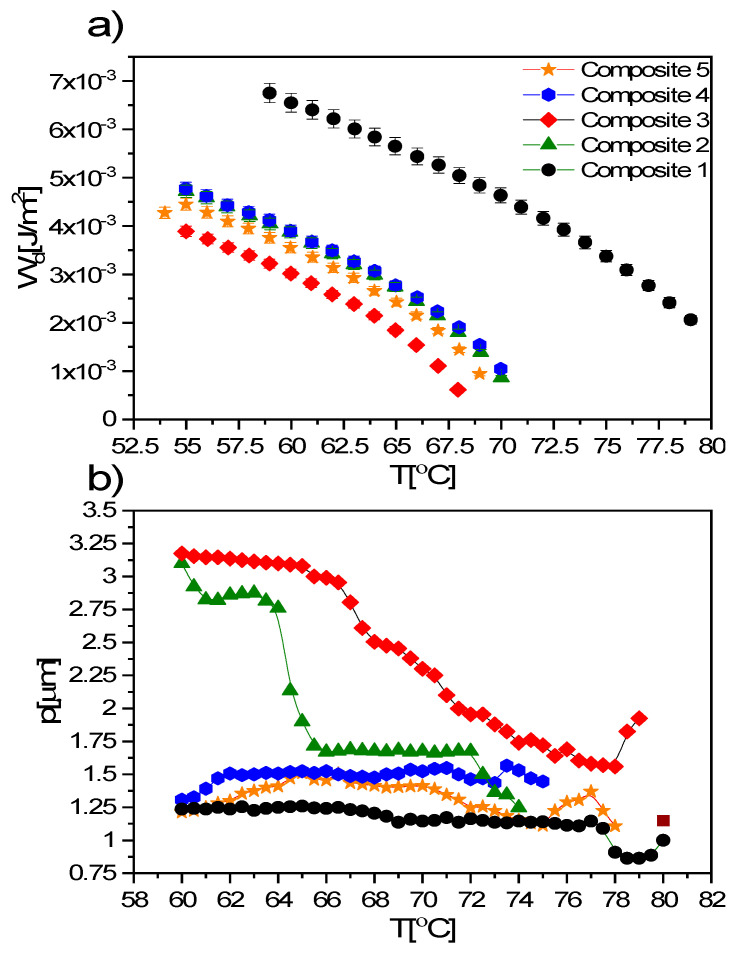
Temperature dependence of the dispersion anchoring coefficient of LC molecules to the surface of the polymer layer (**a**) and temperature dependence of the helix pitch (**b**) for Composites 1–5.

The free energy of ferroelectric liquid crystal contains surface-free energy (additional energy derived from unbalanced forces among the molecules on the surface), which is associated with polar and dispersion contributions [80,81]. Polar anchoring is an electrostatic interaction, while dispersive anchoring is London or van der Waals interactions between LC molecules and the anchoring surface (in our case, a polymer layer) [82,83]. Anchoring energy coefficients, dispersion, *W_d_*, and polar, *W_p_*, can be calculated according to the formulas [80,84,85]:

(2) $$W_{d}\left(T\right)=\frac{\gamma \left(T\right)d}{4\tau \left(T\right)}$$

(3) $$W_{p}\left(T\right)=\sqrt{K\left(T\right)P_{s}\left(T\right)E_{th}\left(T\right)}$$

where *γ*, *d*, *τ*, *K*, *P_s_* and *E_th_* are rotational viscosity, layer thickness, switching time, mean elastic constant, spontaneous polarization and threshold field for molecular reorientation, respectively. Due to the lack of the *K* value, it was not possible to determine the polar anchoring coefficient, *W_p_*. In turn, as shown in Figure 15 the admixture decreases the dispersion coefficient, *W_d_* (mainly due to *γ* and *τ* modification), and its temperature dependence is very similar to the spontaneous polarization (see Figure 11b). Decreasing the *W_d_* coefficient should decrease the threshold voltage for switching between on and off states, which is consistent with the data presented in Figure 11a.

The helix pitch in the SmC* phase is modified by an addition of nanoparticles, as it is shown in Figure 15b. This modification strongly depends on the concentration of nanoparticles used—the lowest is for the highest Fe_2_O_3_ concentration (0.9 wt.%, Composite 5), while the highest is for the lowest concentrations (0.3 and 0.5 wt.%, Composites 2 and 3, respectively). Moreover, deep in the SmC* phase, a continuous increase in the helix pitch was observed for Composite 3, which leads to an increase in the thickness of the smectic layers. It seems that different behavior for lower (0.3 and 0.5 wt.%) and higher (0.7 and 0.9 wt.%) concentrations may arise from the aggregation process. The modification by doping the helix pitch is in good agreement with the tilt angle data for Composites 2 and 3, in contrast to Composite 5 with negligible modification of the helix pitch and significant modification of the tilt angle. The incomprehensible modification of the helix pitch in the case of Composites 4 and 5 (0.7 and 0.9 wt.%) is probably related to poor texture quality, and thus low quality of the fringes, which resulted in underestimating the helix pitch in these nanocomposites. Middha et al. doped cholesteric liquid crystal ZLI–4151 + CB15 using MWCNTs (ca. 110–170 nm, length 5–9 μm, 0.01, 0.1, 0.5, 1.0 wt.%) and obtained materials with shorter helix pitches [86]. Due to hardware limitations, we were unable to determine the helix pitch in the N* phase. It is not excluded that the influence of the admixture (nanoparticles or nanotubes) is quite different for various liquid crystalline phases.

Textures registered for the free drop of Composite 1 (pure EHPDB) in various phases are presented in Supplementary Figure S7. The textures of isotropic liquid and the SmA* phase are completely black (Figure S7a,d), while characteristic textures of cholesteric N* phase are visible in Supplementary Figure S7b. In turn, the common fingerprint texture of the TGBA* phase is clearly visible in Supplementary Figure S7c. Interestingly, the other fingerprint texture (Supplementary Figure S7e,f) was also registered below the SmA* phase, and we suppose that it is another frustrated phase in a very narrow temperature range, e.g., TGBC* (not registered by other methods). Characteristic textures of a chiral ferroelectric SmC* phase with an equidistant line pattern are presented in Supplementary Figure S7f,g. We suppose that detection of the frustrated TGBC* phase was possible in this study due to the very slow cooling rate (0.1 °C/min) not used in other methods (5 °C/min in DSC or 3 °C/min in TLI), but a thorough investigation of the presence of this phase in nanocomposites is not the aim of this study.

### 3.9. FTIR Results

**Figure 16:**
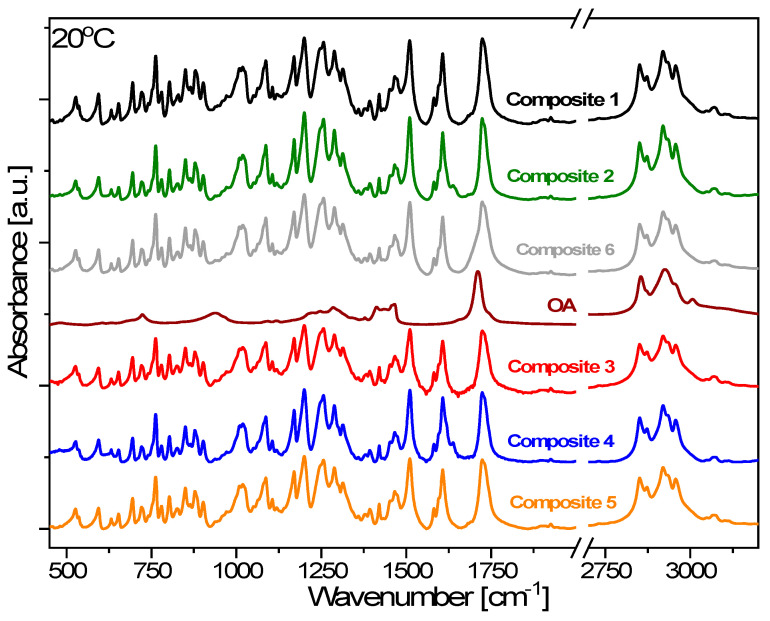
FTIR spectra of Composites 1–6 and oleic acid obtained at 20 °C.

The comparison of FTIR spectra obtained at 20 °C for Composites 1–6 and oleic acid is presented in Figure 16. For better visibility, two spectral ranges: 450–2000 and 2700–3200 cm^−1^, where all fundamental bands occur, are exposed. The IR spectrum of Composite 1 (pure EHPDB) fully confirms its proper chemical and molecular composition—all the expected essential bands are present in the spectrum. Most prominent bands are connected to ν(C–O) and ν(C=O) stretching modes in aromatic ester. The first occurred in the spectral range of 1050–1300 cm^−1^ and the second one was placed at 1724 cm^−1^. Additionally, very intensive bands connected to stretching ν(CH)_ar_ and bending δ_oop_(CH)_ar_ vibrations within para-substituted aromatic rings appeared at 1607, 1510 and 850 cm^−1^, respectively. The band characteristic for the epoxy ring appeared at 902 cm^−1^ and is connected to ν(C–O)_epoxy_. The spectrum also contains stretching and bending vibrations of methyl and methylene groups of aliphatic chain, which occurred in the spectral ranges of 2800–3000 and 1300–1480 cm^−1^, respectively.

Comparing the spectra of Composite 1 and nanocomposites (Composites 2–5), it can be seen that they are basically identical taking into consideration positions and intensities of all bands. Additionally, no new bands were observed in the spectra of all composites, which suggests that there are no molecular interactions between Fe_2_O_3_ nanoparticles and EHPDB that would lead to modification of the chemical structure of the liquid crystal matrix. However, it has to be noted that the amount of Fe_2_O_3_ nanoparticles in the investigated materials is below 1 wt.%, and at such a low concentration, the IR method may not be sensitive enough to see it. In case of Composites 2 and 4, one additional band with a maximum at 1638 cm^−1^ can be seen, which is connected to a small amount of water impurity in these samples. On heating the samples up to 100 °C, the band disappeared due to its evaporation (see Supplementary Figure S8). Comparing the spectra of Composite 2 and Composite 6, one can see that they are also basically the same, except for the region between 1670 and 1710 cm^−1^, where some broadening is clearly visible in case of the Composite with oleic acid. It comes from the band connected to the stretching vibration of the carbonyl group, which is most prominent in the spectrum of oleic acid. It proves that oleic acid is present in this sample, but it does not change the molecular structure of the investigated composite as we did not observe any changes in bands’ positions or intensities. In turn, the spectra of Composites 1–6 obviously changed upon heating to 100 °C, but again, there were no differences between them (see Supplementary Figure S8a), while the spectra obtained at 10 °C, after the heating–cooling cycle (see Supplementary Figure S8b), are comparable to the ones obtained for the samples without thermal history and being in the ordered crystalline phase (Figure 16).

## 4. Conclusions

In this paper, we presented the effect of low concentrations of a γ-Fe_2_O_3_ nanoparticles admixture (containing small amounts of hematite and magnetite) on the physical properties of the EHPDB ferroelectric liquid crystal, especially in the SmC* phase. The influence of the addition of oleic acid to the nanocomposites was also studied. A number of complementary methods were used, and based on the obtained results, the following conclusions can be drawn:

- The structure of the EHPDB matrix did not change after doping. The diffraction patterns of the nanocomposites are practically the same as in the case of pure EHPDB, only the low-intensity reflections coming from Fe_2_O_3_ nanoparticles exist in addition. The surface morphology of the nanocomposites also did not change after doping (without oleic acid).
- The following phases during cooling were registered for studied composites: BP, N*, TGBA*, SmA*, TGBC* and SmC*. The shift of the phase transition temperatures to lower values, the disappearance of BP and the polymorphism of crystal phases, as well as the broadening of the SmA* versus the narrowing of the SmC* phase after the addition of Fe_2_O_3_ nanoparticles, were observed. What is interesting is that a very narrow TGBA* phase existed in all nanocomposites. The broadening of the SmA* phase in favor of SmC* was explained by modifying the mobility of nanoparticles. A very strong influence of the admixture on the texture of the SmC* phase in nanocomposites was observed as a strong striation.
- A very strong influence of the admixture on the electrical parameters was noted. The spontaneous polarization, tilt angle, switching time and rotational viscosity decreased after doping. The additional voltage response registered for nanocomposites was assigned to the additional ions introduced with nanoparticles into the EHPDB matrix. A slightly different modification of the SmC * phase parameters for nanocomposites with higher concentrations of nanoparticles (0.7 and 0.9 wt.%) was explained by the strong influence of Fe_2_O_3_ aggregation.
- The addition of oleic acid greatly narrowed the range of the mesophases with cooling and practically caused the disappearance of the liquid crystal polymorphism during heating. It was also responsible for the formation of ripples on the surface and changing the orientation of organic molecules from planar to homeotropic in a quite specific way (depending on the diameter of cholesteric droplets).

To summarize, the aim of our work was to show that materials with precisely defined parameters can be obtained by controlling the concentration of a nanoparticle admixture. As we have shown, modification of the electrical parameters of the LC matrix by the nanoparticles (at very small concentrations) is significant, however the explanation is not easy; very often, several effects may modify a given property of the matrix. It should be kept in mind that the interactions: LC molecule–LC molecule, LC molecule–nanoparticle, nanoparticle–nanoparticle and nanoparticle–electrode, should also be considered. Therefore, the issue is complex and requires further experimental research or advanced computer simulations.

## Figures and Tables

**Table 1:** Mass compositions of Composites 1–6 and Fe_2_O_3_ samples with and without oleic acid.

Composite	Amount of EHPDB	Amount of Fe_2_O_3_	Amount of Oleic Acid
Composite 1 (0.0 wt.%)	40.52 mg	0.00 mg	–
Composite 2 (0.3 wt.%)	40.32 mg	0.12 mg	–
Composite 3 (0.5 wt.%)	40.20 mg	0.20 mg	–
Composite 4 (0.7 wt.%)	40.91 mg	0.29 mg	–
Composite 5 (0.9 wt.%)	40.20 mg	0.37 mg	–
Composite 6 (0.3 wt.% + OA)	40.07 mg	0.12 mg	0.5 μL
	**Amount of Fe_2_O_3_**		**Amount of Oleic Acid**
Sample 1	36.00 mg 36.01 mg		–
Sample 2			150.0 μL

## Data Availability

Not applicable.

## Supplementary Materials

The following are available online at https://www.mdpi.com/article/10.3390/ma14164722/s1, Figure S1: Digital optical images of Sample 1, Sample 2 and Composite 1. The magnification is shown in the images, where colored dotted squares correspond to the enlarged areas, Figure S2: SEM images of Samples 1–2 and Composites 1–6. The magnification is shown in the images, where colored dotted squares correspond to the enlarged areas, Figure S3: DSC curves registered during heating at a rate of 10 °C/min for Composites 1–6 (a) and extended temperature range of 75.0–100.0 °C (b), Figure S4: Textures of the N* phase registered for Composite 3 during cooling down from isotropic phase (2 °C/min). Formation of N* phase droplets immediately after the Is–N* phase transition with visible focal-conic inclusions (a). Typical fan-like texture (b). Cracking of fan-like structure (c). Beginning of forming stripes on a cracked fan-shape structure (d). The formation process of streaks characteristic of the oily-streaks texture (e). Typical oily-streaks texture from which a transition to the TGBA* frustrated phase was observed at lower temperatures (f). All images show the same sample area as the temperature is lowered, Figure S5: Influence of Fe_2_O_3_ nanoparticles admixture on the phase transitions during heating: Cr–SmC*, SmC*–SmA*, N*–Is/P–N*, Figure S6: The alignment process of Composites 1–5 (20 V_pp_, 50 Hz, rectangular wave), Figure S7: Textures registered for the free drop of Composite 1 (pure EHPDB) in: isotropic phase, 96.1 °C (a), cholesteric phase, 90.5 °C (b), TGBA* phase, 84.0 °C (c), SmA* phase, 82.5 °C (d), TGBC* phase, 80.0 °C (e), TGBC* phase, 79.50 °C (f), SmC* phase, 78.5 °C (g), SmC* phase, 60.0 °C (h), Figure S8: FTIR spectra of Composites 1–6 and oleic acid obtained at 100 °C (a) and 10 °C, after the heating–cooling cycle (b), Table S1: The phase sequences on heating and cooling for Composites 1–6. The TGBA*–SmA* phase transition temperature was omitted here due to the large uncertainties in determination.
