# Supplementary material for: Modifications of FLC Physical Properties through Doping with Fe2O3 Nanoparticles (Part I)
Source: Materials (Basel). 2021 Aug 21;14(16):4722. doi: 10.3390/ma14164722 (PMC8399294; doi:10.3390/ma14164722)
Supplement: Supplementary file 1 [file materials-14-04722-s001.zip › materials-1346106-supplementary.pdf]

materials

MDPI

Supplementary Information

Modifications of FLC Physical Properties through Doping with Fe_2O_3 Nanoparticles (Part I)

1. Sample preparation

The Composite 6 was prepared according to the scheme:

1. The adequate amounts of pure EHPDB and Fe_2O_3 nanoparticles were weighed into separate vials (see Table 1).
2. Oleic acid of 0.5 μL was added to vial with nanoparticles at room temperature (a small drop of oleic acid was spread on the bottom of vial to cover the nanoparticles) and the vial was left at room temperature for several hours.
3. 1.5 mL of chloroform was added to the nanoparticles + oleic acid at room temperature and the solution was sonicated in 50 s(on)/10 s(off) mode for 45 min (A = 30%, ice blanket).
4. Steps like for Composites 1–5.

Two samples of Fe_2O_3 nanoparticles was prepared to check the influence of oleic acid on the properties of nanoparticles: pure nanoparticles (Sample 1) and nanoparticles decorated with oleic acid (Sample 2):

Sample 1: The appropriate amount of nanoparticles was weighed.

Sample 2: The appropriate amount of nanoparticles was weighed into vial and oleic acid of 150.0 μL was spread on the bottom of vial to cover the nanoparticles. The vial was left for several hours. Next, the steps 3–9 of the synthesis route of Composite 6 (without the steps related to EHPDB) were done, except the time in step 6 was 225 min in order to obtain about 1mL of solution.

2. Results

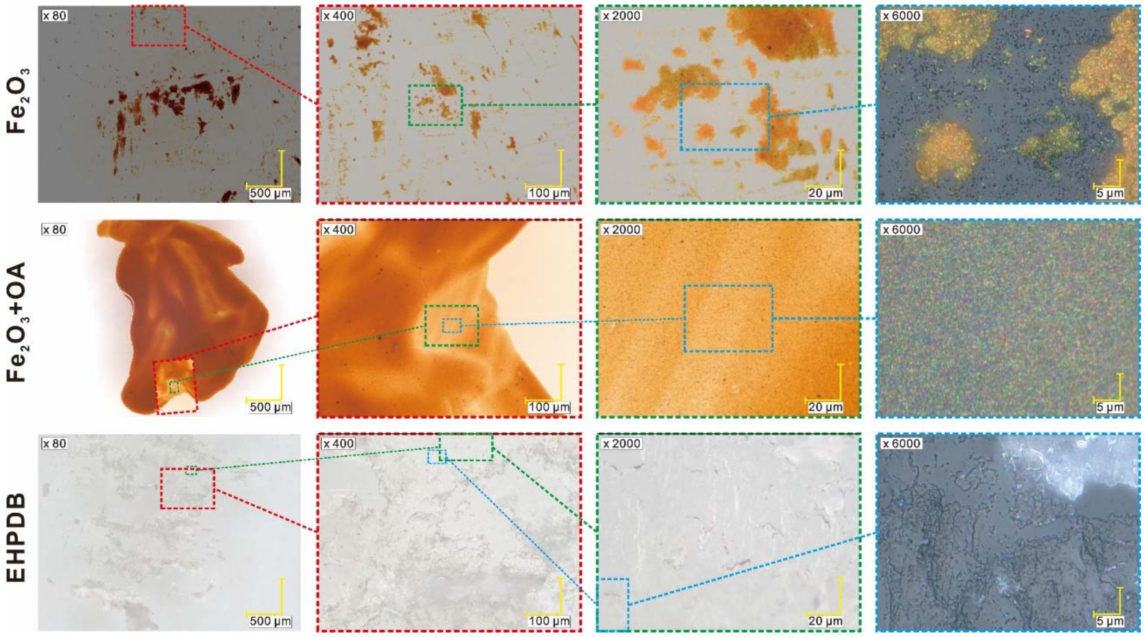

Figure S1. Digital optical images of Sample 1, Sample 2 and Composite 1. The magnification is shown in the images, coloured dotted squares correspond to the enlarged areas.

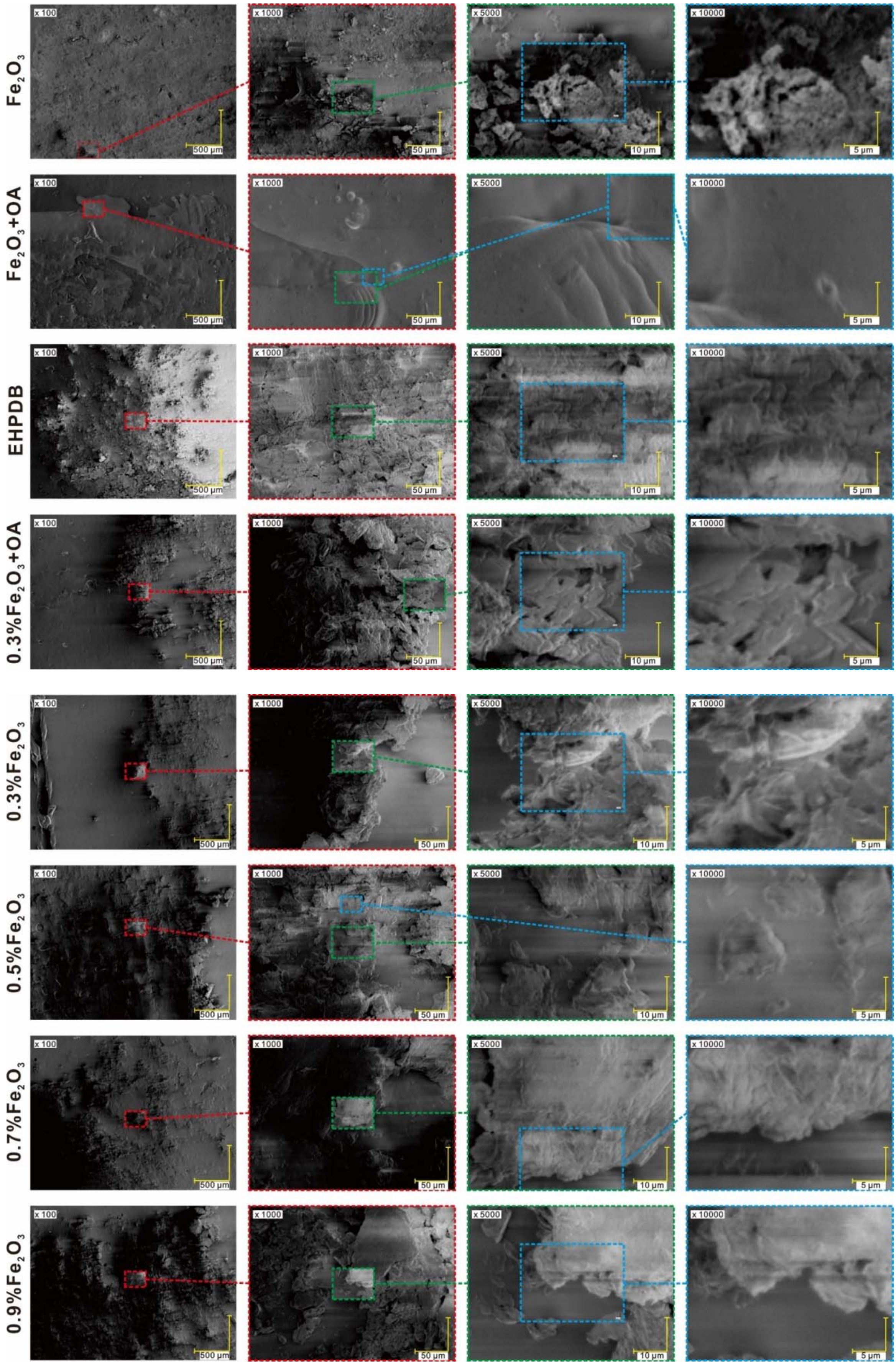

Figure S2. SEM images of Samples 1–2 and Composites 1–6. The magnification is given in the images, coloured dotted squares correspond to the enlarged areas.

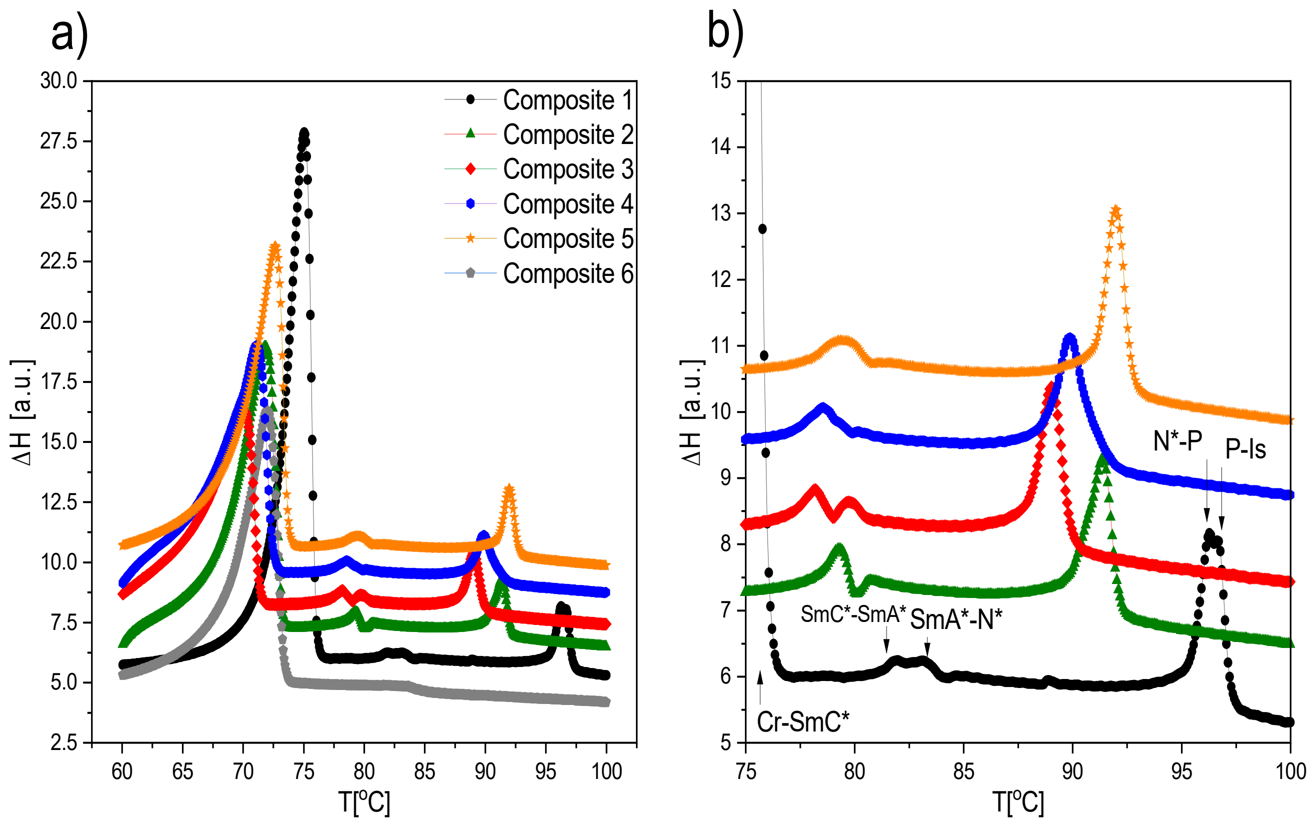

Figure S3. DSC curves registered during heating at 10 °C/min rate for Composites 1–6 (**a**) and extended temperature range 75.0–100.0 °C (**b**).

In Figure S3 the first anomaly represents the transition to the ferroelectric phase, the second transition between SmC*–SmA*, the third paraelectric–cholesteric phase transition and the last one N*–isotropic liquid phase transition. As can be seen the transition SmA*–N* for the Composite 4 is visible in the form of a shoulder while for the Composite 5 we observe two connected transitions into one with a wide width–half. In the case of a Composite 1 the last phase transition is double, which indicates a narrow range of the extra phase which was also mentioned in cooling cycle. A small hump visible around 89.0 °C for Composite 1 it is an artefact, not observed for measurements at other rates. The admixture used in a concentration of 0.5 wt.% causes a significant narrowing of the cholesteric phase, which was also weakened during cooling.

One of the most commonly observed textures for the N* phase is the oily–streaks texture. For planar conditions, in N* phase the long molecular axis lies within the smectic plane which is perpendicular to the plates. This implies that the cholesteric axis of the helix is oriented parallel to the glass plates. In the case of the Composite 1, the cholesteric phase was immediately formed into an oily–streaks texture. The oily–streaks texture represents a network of defect lines immersed in homogeneously helical regions. The structure of oily–streaks depends, inter alia, on surface anchoring. In general, the oily–streaks network is not static, but it can grow or fade, resulting, for example, in a uniformly oriented sample. This behavior was observed for the pure matrix (the oily–streaks texture changes relatively quickly to a uniform color—the Grandjean texture due to lowering the temperature). Moreover, the observed streaks in the pure matrix were single. Completely different behavior was observed in the case of all composites. Another natural texture seen in the cholesteric phase is fan–shaped texture. From this texture, the N* phase began to form in all nanocomposites.

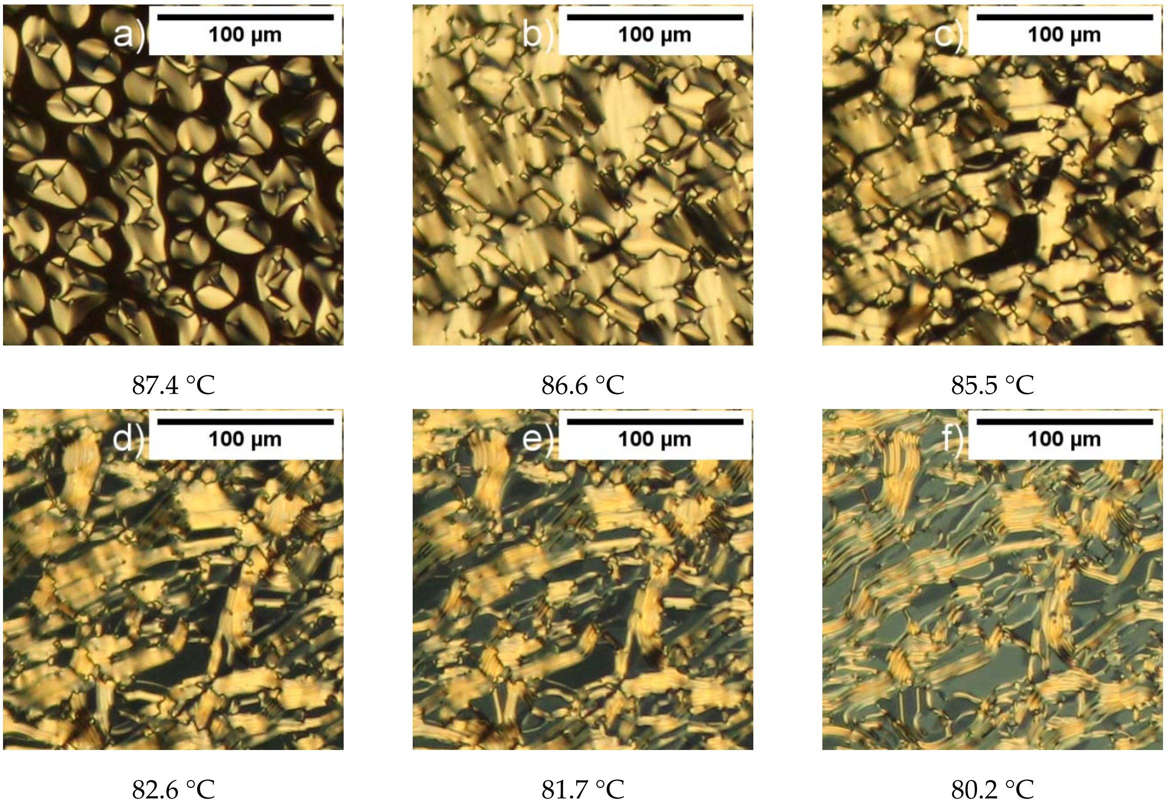

Figure S4. Textures of the N* phase registered for Composite 3 during cooling down from isotropic phase (2 °C/min). Formation of N* phase droplets immediately after the Is–N* phase transition with visible focal–conic inclusions (**a**). Typical fan–like texture (**b**). Cracking of fan–like structure (**c**). Beginning of forming stripes on a cracked fan–shape structure (**d**). The formation process of streaks characteristic for the texture oily–streaks (**e**). Typical oily–streaks texture from which a transition to the TGBA* frustrated phase was observed at lower temperatures (**f**). All images show the same sample area as the temperature is lowered.

Table S1. The phase sequences on heating and cooling for Composites 1–6. The TGBA*–SmA* phase transition temperature was omitted here due to the large uncertainties in determining.

**Composites**	**The Phase Sequence (Cooling)**
Composite 1	$Is \xrightarrow{\text{96.29°C(0.01°C)}} P \xrightarrow{\text{95.11°C}} N^* \xrightarrow{\text{83.19°C(0.20°C)}} TGBA^* \xrightarrow{\text{\textasciitilde 80.06°C}} SmA^* \xrightarrow{\text{\textasciitilde 79.00°C}} SmC^* \xrightarrow{\text{58.07°C(0.04°C)}} Cr_1 \xrightarrow{\text{56.93°C}} Cr_2 \xrightarrow{\text{55.69°C}} Cr_3 \xrightarrow{\text{54.73°C}} Cr_4 \xrightarrow{\text{44.64°C(0.18°C)}} Cr_5 \xrightarrow{\text{41.25°C}} Cr_6 \xrightarrow{\text{40.43°C}} Cr_7$
Composite 2	$Is \xrightarrow{\text{91.24°C(0.03°C)}} (P)N^* \xrightarrow{\text{79.42°C(0.13°C)}} (TGBA^*)SmA^* \xrightarrow{\text{72.20°C}} SmC^* \xrightarrow{\text{55.79°C(0.04°C)}} Cr_1 \xrightarrow{\text{54.58°C}} Cr_2 \xrightarrow{\text{38.31°C(0.13°C)}} Cr_3$
Composite 3	$Is \xrightarrow{\text{89.10°C(0.30°C)}} N^* \xrightarrow{\text{79.85°C(0.06°C)}} (TGBA^*)SmA^* \xrightarrow{\text{67.70°C}} SmC^* \xrightarrow{\text{53.48°C}} Cr_1 \xrightarrow{\text{52.75°C}} Cr_2 \xrightarrow{\text{51.65°C}} Cr_3 \xrightarrow{\text{50.77°C}} Cr_4 \xrightarrow{\text{50.18°C}} Cr_5 \xrightarrow{\text{49.45°C}} Cr_6 \xrightarrow{\text{34.63°C(0.14°C)}} Cr_7$
Composite 4	$Is \xrightarrow{\text{89.63°C(0.13°C)}} (P)N^* \xrightarrow{\text{78.51°C(0.19°C)}} (TGBA^*)SmA^* \xrightarrow{\text{69.50°C}} SmC^* \xrightarrow{\text{54.61°C(0.10°C)}} Cr_1 \xrightarrow{\text{36.10°C(0.15°C)}} Cr_2$
Composite 5	$Is \xrightarrow{\text{91.91°C(0.02°C)}} N^* \xrightarrow{\text{80.09°C(0.06°C)}} (TGBA^*)\ SmA^* \xrightarrow{\text{69.80°C}} SmC^* \xrightarrow{\text{55.56°C(0.40°C)}} Cr_1 \xrightarrow{\text{52.52°C}} Cr_2 \xrightarrow{\text{37.95°C(0.20°C)}} Cr_3$
Composite 6	$Is \xrightarrow{\text{73.21°C}} X \xrightarrow{\text{72.09°C(0.36°C)}} N^* \xrightarrow{\text{62.64°C(0.83°C)}} SmA^* \xrightarrow{\text{52.36°C(0.05°C)}} SmC^* \xrightarrow{\textbf{50.76}\text{°C}} Cr_1 \xrightarrow{\text{39.84°C(0.19°C)}} Cr_2$
**Composites**	**The Phase Sequence (Heating)**
Composite 1	$Cr_1 \rightarrow Cr_2 \rightarrow Cr_3 \xrightarrow{\text{72.51°C(0.11°C)}} SmC^* \xrightarrow{\text{\textasciitilde 78.00°C}} TGBC^* \xrightarrow{\text{80.76°C(0.10°C)}} SmA^* \xrightarrow{\text{82.04°C(0.20°C)}} TGBA^* \xrightarrow{\text{\textasciitilde 84.00°C}} N^* \xrightarrow{\text{94.92°C(0.04°C)}} P \xrightarrow{\text{96.63°C}} Is$
Composite 2	$Cr \xrightarrow{\text{68.28°C(0.12°C)}} SmC^* \xrightarrow{\text{\textasciitilde 76.00°C}} TGBC^* \xrightarrow{\text{77.72°C(0.05°C)}} SmA^* \xrightarrow{\text{79.34°C(0.14°C)}} N^* \xrightarrow{\text{89.67°C(0.09°C)}} Is$
Composite 3	$Cr \xrightarrow{\text{65.62°C(0.65°C)}} SmC^* \xrightarrow{\text{76.38°C(0.13°C)}} SmA^* \xrightarrow{\text{78.69°C(0.10°C)}} N^* \xrightarrow{\text{87.45°C(0.07°C)}} Is$
Composite 4	$Cr \xrightarrow{\text{66.84°C(0.35°C)}} SmC^* \xrightarrow{\text{76.97°C(0.26°C)}} SmA^* \xrightarrow{\text{\textasciitilde 79.00°C}} N^* \xrightarrow{\text{88.22°C(0.12°C)}} Is$
Composite 5	$Cr \xrightarrow{\text{69.06°C(0.14°C)}} SmC^* \xrightarrow{\text{77.73°C(0.07°C)}} SmA^* \xrightarrow{\text{\textasciitilde 79.00°C}} N^* \xrightarrow{\text{90.78°C(0.02°C)}} Is$
Composite 6	$Cr_2 \xrightarrow{\text{69.48°C(0.11°C)}} Cr_2 + N^* \xrightarrow{\text{75.57°C(7.77°C)}} Is$

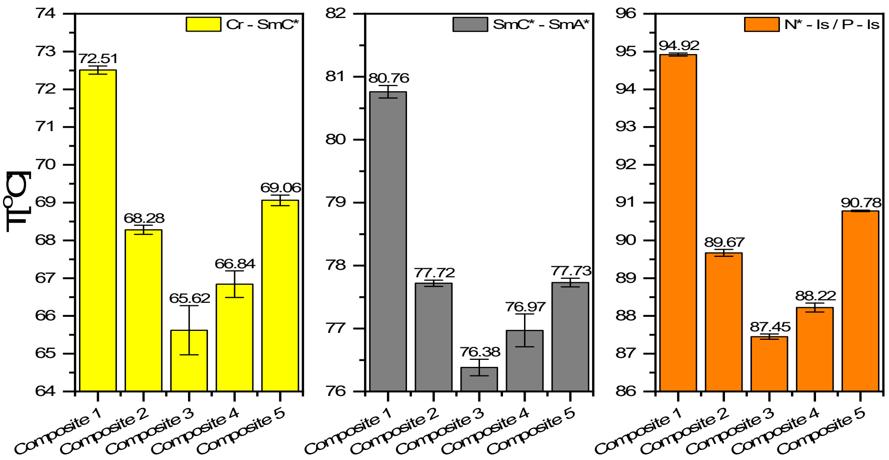

Figure S5. Influence of Fe_2O_3 nanoparticles admixture on the phase transitions during heating: Cr–SmC*, SmC*–SmA*, N*–Is/P–N*.

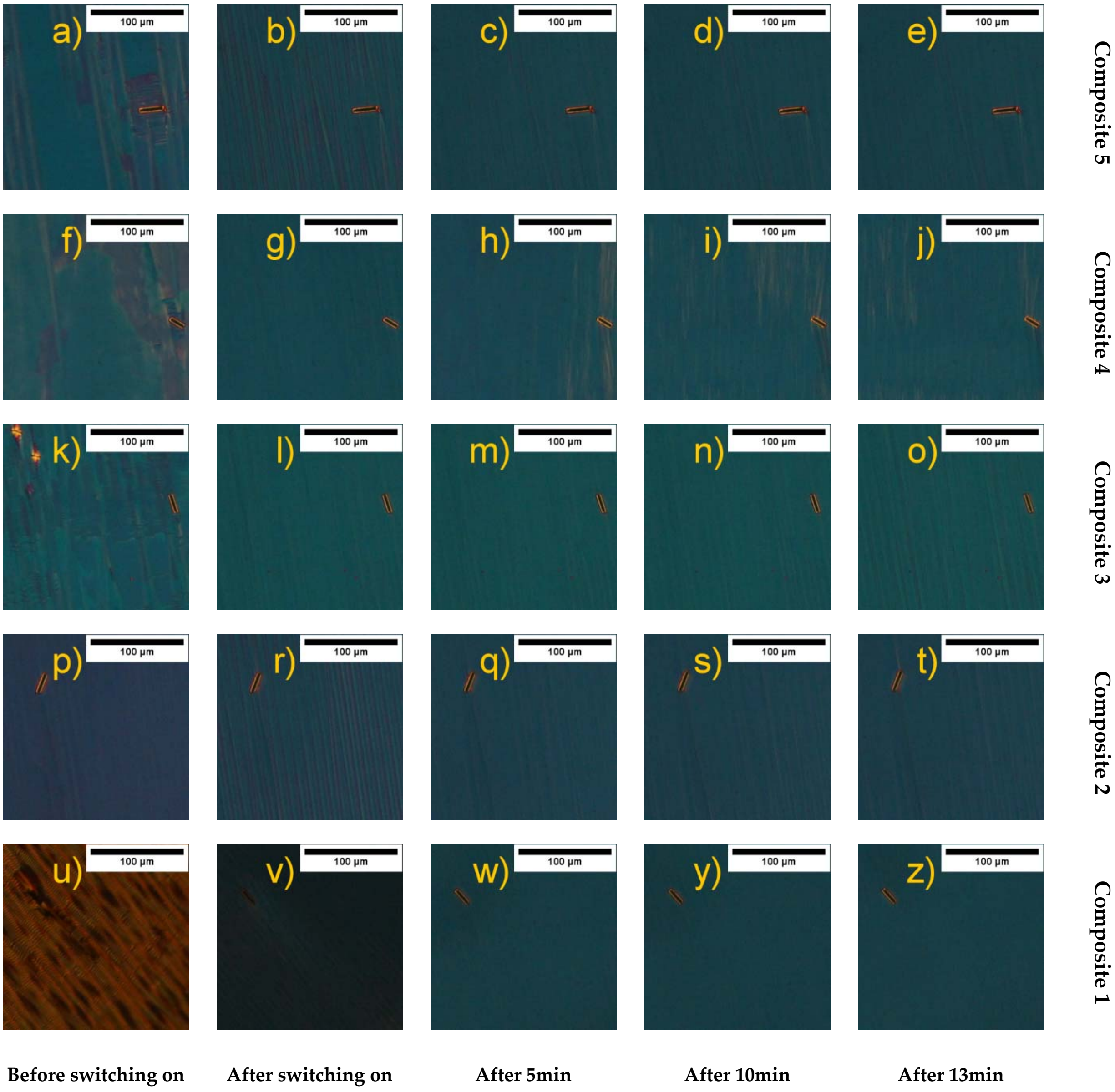

Figure S6. The alignment process of Composites 1–5 ($20V_{PP}$, 50 Hz, rectangular wave).

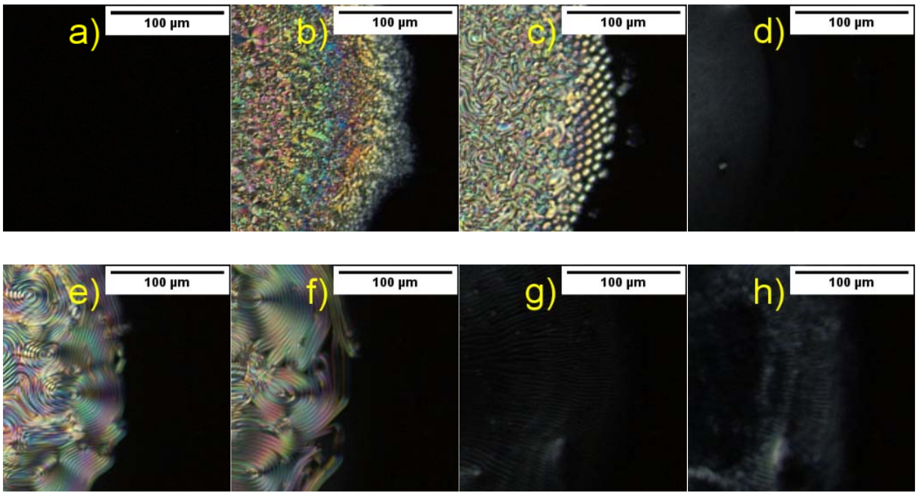

Figure S7. Textures registered for the free drop of Composite 1 (pure EHPDB) in: isotropic phase, 96.1 °C (**a**); cholesteric phase, 90.5 °C (**b**); TGBA* phase, 84.0 °C (**c**); SmA* phase, 82.5 °C (**d**); TGBC* phase, 80.0 °C (**e**); and TGBC* phase, 79.50 °C (**f**); SmC* phase, 78.5 °C (**g**); SmC* phase, 60.0 °C (**h**).

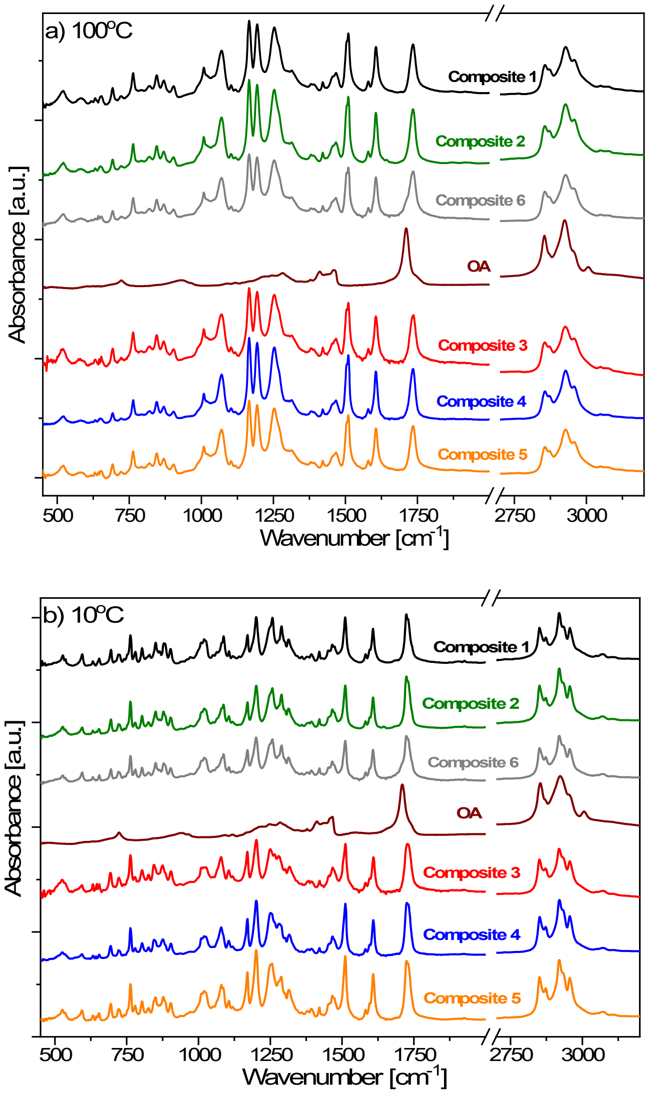

Figure 8. FTIR spectra of Composite 1–6 and oleic acid obtained at 100 °C (**a**) and 10 °C, after the heating–cooling cycle (**b**).

The comparison of the spectra of Composites 1–6 obtained after heating to 100°C, thus in the isotropic phase, is presented in Figure S7a. Additionally, the spectrum of oleic acid is shown at this temperature for comparison. The spectra of all Composites 1–6 obviously change upon heating but again there are no differences between them. The broadening of the band connected to vibration of carbonyl group in the spectrum of the composite with oleic acid is still visible. In turn, in Figure S7b the spectra obtained for all Composites 1–6 with pure oleic acid at 10 °C (after heating–cooling cycle) are shown. They are comparable to the ones obtained for the samples without thermal history and being in the ordered crystalline phase (Figure 17). Some discrepancies between the spectra obtained at 10 °C can be noticed in the spectral ranges of: 835–893, 1050–1100 and 1220–1340 cm^{-1}. This may be due to the fact that the transition from the liquid crystal to ordered crystalline phase does not occur in its entirety.
